# On Computability and Triviality of Well Groups

**DOI:** 10.1007/s00454-016-9794-2

**Published:** 2016-05-31

**Authors:** Peter Franek, Marek Krčál

**Affiliations:** IST Austria, Am Campus 1, 3400 Klosterneuburg, Austria

**Keywords:** Nonlinear equations, Robustness, Well groups, Computational topology, Obstruction theory, Homotopy theory, 65H10, 68U05, 55S35, 55Q55

## Abstract

The concept of *well group* in a special but important case captures homological properties of the zero set of a continuous map $f:K\rightarrow {\mathbb {R}}^n$ on a compact space *K* that are invariant with respect to perturbations of *f*. The perturbations are arbitrary continuous maps within $L_\infty$ distance *r* from *f* for a given $r>0$. The main drawback of the approach is that the computability of well groups was shown only when $\dim K=n$ or $n=1$. Our contribution to the theory of well groups is twofold: on the one hand we improve on the computability issue, but on the other hand we present a range of examples where the well groups are incomplete invariants, that is, fail to capture certain important robust properties of the zero set. For the first part, we identify a computable subgroup of the well group that is obtained by cap product with the pullback of the orientation of ${\mathbb {R}}^n$ by *f*. In other words, well groups can be algorithmically approximated from below. When *f* is smooth and $\dim K<2n-2$, our approximation of the $(\dim \ K-n)$th well group is exact. For the second part, we find examples of maps $f,f':K\rightarrow {\mathbb {R}}^n$ with all well groups isomorphic but whose perturbations have different zero sets. We discuss on a possible replacement of the well groups of vector valued maps by an invariant of a better descriptive power and computability status.

## Introduction

In many engineering and scientific solutions, a highly desired property is the resistance against noise or perturbations. We can only name a fraction of the instances: stability in data analysis [7], robust optimization [3], image processing [17], or stability of numerical methods [19]. Some very important tools for robust design come from topology, which can capture stable properties of spaces and maps.

In this paper, we take the robustness perspective on the study of the solution set of systems of nonlinear equations, a fundamental problem in mathematics and computer science. Equations arising in mathematical modeling of real problems are usually inferred from observations, measurements or previous computations. We want to extract maximal information about the solution set, if an estimate of the error in the input data is given.

More formally, for a continuous map $f:K\rightarrow {\mathbb {R}}^n$ on a compact Hausdorff space *K* and $r>0$ we want to study properties of the family of zero sets

$$\begin{aligned} Z_r(f):=\{g^{-1}(0):\Vert f-g\Vert \le r\}, \end{aligned}$$

where $\Vert \cdot \Vert$ is the max-norm with respect to some fixed norm $|\cdot |$ in ${\mathbb {R}}^n$. The functions *g* with $\Vert f-g\Vert \le r$ (or $\Vert f-g\Vert <r$) will be referred to as *r*-*perturbations of* *f* (or *strict* *r*-*perturbations of* *f*, respectively). Quite notably, we are not restricted to *parameterized* perturbations but allow arbitrary continuous functions at most (or less than) *r* far from *f* in the max-norm.

*Well Groups.* Recently, the concept of well groups was designed to quantify transversality of a map $K\rightarrow Y$ with respect to a subspace $Y'$ of *Y*. Following [11], we assume^1^ a subspace ${\mathcal {P}}$ of *C*(*K*, *Y*) with a metric *d*. Any $h\in {\mathcal {P}}$ for which $d(h,f)\le r$ is called an *r*-*perturbation* of *f* and we define $X_r\subseteq K$ to be the union of $g^{-1}(Y')$ over all *r*-perturbations *h* of *f*. The inclusion $h^{-1}(Y')\hookrightarrow X_r$ induces a map in homology $j_h: H(h^{-1}(Y'))\rightarrow H(X_r)$, where *H* is a suitable homology theory. For $r>0$, the well group *U*(*r*) is defined to be the intersection $\cap \,\mathrm {Im}(j_h)$ over all *r*-perturbations *h* of *f*. Informally, well group capture the robustness of intersection of *f*(*K*) with $Y'$ via means of homology, namely “homological properties” that are common to all $h^{-1}(Y')$ for all *r*-perturbations *h* of *f*.

We will deal with the special but important case when $Y={\mathbb {R}}^n$, $Y'=\{0\}$, and ${\mathcal {P}}$ is the space of all continuous functions $C(K,{\mathbb {R}}^n)$ with the max-norm with respect to a fixed norm on ${\mathbb {R}}^n$. Then well group is a property of $Z_r(f)$ that captures homological properties common to all *zero sets* in $Z_r(f)$. We enhance the theory to include a *relative case* 2 that is especially convenient in the case when *K* is a manifold with boundary. Let $B\subseteq K$ be a pair of compact Hausdorff spaces and $f: K\rightarrow {\mathbb {R}}^n$ continuous. Let $X:=|f|^{-1} [0,r]$ where |*f*| denotes the function $x\mapsto |f(x)|$; this is the smallest space containing zero sets of all *r*-perturbations *h* of *f* and coincides with $X_r$ from the notation of [11].

In the rest of the paper, for any space $Z\subseteq K$ we will abbreviate the pair $(Z,Z\cap B$) by (*Z*, *B*) and, similarly for homology, $H_*(Z,Z\cap B)$) by $H_*(Z,B)$. Everywhere in the paper we use homology and cohomology groups with coefficients in ${\mathbb {Z}}$ unless explicitly stated otherwise. For brevity we omit the coefficients from the notation.

**Fig. 1:**
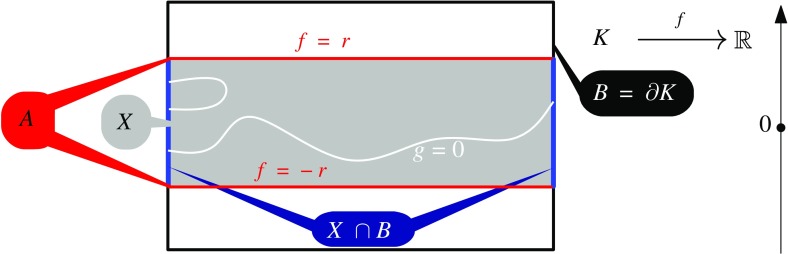
For the projection $f(x,y)=y$ to the vertical axis defined on a box *K*, the zero set of every *r*-perturbation is contained in $X=|f|^{-1}[0,r]$ and $\partial X$ consists of *A* (upper and lower side) where $|f|=r$, and $X\cap B\subseteq \partial K$. The zero set always separates the two components of *A*. On the homological level, the zero set “connects” the two components of $X\cap B$ and the image of $H_1(g^{-1}(0),B)$ in $H_1(X,B)$ is always surjective and thus $U_1(f,r)\cong H_1(X,B)$. Note that the well group would be trivial with $B=\emptyset$

We define the *j*th well group $U_j(f,r)$ of *f* at radius *r* as the subgroup of $H_j(X,B)$

$$\begin{aligned} U_j(f,r):=\bigcap _{Z\in Z_r(f)} \, \mathrm {Im}\big (H_j(Z, B)\mathop {\longrightarrow }\limits ^{i_*} H_j(X,B)\big ), \end{aligned}$$

where $i_*$ is induced by the inclusion $i:Z \hookrightarrow X$ and *H* refers to a convenient homology theory of compact metrizable spaces that we describe below.^3^ This reduces to the original definition whenever $B=\emptyset$. For a simple example of a map *f* with nontrivial first well group see Fig. 1 on page 5.

*Significance of Well Groups.* We only mention a few of many interesting things mostly related to our setting. The well group in dimension zero characterizes robustness of solutions of a system of equations $f(x)=0$. Namely, $\emptyset \in Z_r(f)$ if and only if $U_0(f,r)\cong 0$. Higher well groups capture additional robust topological properties of the zero set such as in Fig. 1. Perhaps the most important is their ability to form *well diagrams* [11]—a kind of measure for robustness of the zero set (or more generally, robustness of the intersection of *f* with other subspace $Y'\subseteq Y)$. The well diagrams are stable with respect to taking perturbations of *f*.^4^

*Homology Theory.* For the foundation of well groups we need a homology theory on compact Hausdorff spaces that satisfies some additional properties that we specify later in Sect. 3. Roughly speaking, we want that the homology theory behaves well with respect to infinite intersections. Without these properties we would have to consider only “well behaved” perturbations of a given *f* in order to be able to obtain some nontrivial well groups in dimension greater than zero. We explain this in more detail also in Sect. 3. For the moment it is enough to say that the *Čech* homology can be used and that for any computational purposes it behaves like simplicial homology. In Sect. 3 we explain why using singular homology would make the notion of well groups trivial.

A basic ingredient of our methods is the notion of *cap product*

$$\begin{aligned} \frown : H^n(X,A)\otimes H_k(X,A\cup B)\rightarrow H_{k-n}(X,B) \end{aligned}$$

between cohomology and homology. We refer the reader to [28, Sect. 2.2] and [18, p. 239] for its properties and to Appendix 4 for its construction in Čech (co)homology. Again, it behaves like the simplicial cap product when applied to simplicial complexes. For an algorithmic implementation, one can use its simplicial definition from [28].

### Computability Results

The main result introduced in this section is the computability of certain subgroup of the well group that in many cases coincides with the full well group. A core ingredient is certain cohomology class that we will call *primary obstruction*. Intuitively, it assigns to each *n*-cell in a triangulation of *X* the intersection number with arbitrary zero set in $Z_r(f)$. In the example illustrated in Fig. 1, it would be an element assigning $\pm 1$ to each vertical edge of a triangulation of the rectangle *X* that connects the “lower face” with the “upper face”, indicating that such edge intersects the zero set at least once. If *X* is a compact oriented connected manifold, there is a well-defined *Poincaré dual* of the primary obstruction: this is a homology class of a cycle that intersects the cells of *X* as prescribed by the primary obstruction. We will show that not only the Poincaré dual but, even more generally, any *cap* product^5^ of the primary obstruction is contained in the well group of *f*.

*Computer Representation.* To speak about computability, we need to fix some computer representation of the input. Here we assume the simple but general setting of [14], namely, *K* is a finite simplicial complex, $B\subseteq K$ a subcomplex, *f* is simplexwise linear with rational values on vertices^6^ and the norm $|\cdot |$ in ${\mathbb {R}}^n$ can be (but is not restricted to) $\ell _1,\ell _2$ or $\ell _\infty$ norm.

*Previous Results.* The algorithm for the computation of well groups was developed only in the particular cases of $n=1$ [4] or $\dim K=n$ [8]. In [14] we settled the computational complexity of the well group $U_0(f,r)$. The complexity is essentially identical to deciding whether the restriction $f|_A:A\rightarrow S^{n-1}$ can be extended to $X\rightarrow S^{n-1}$ for $A=|f|^{-1}(r)$, or equivalently, $A=f^{-1}(S^{n-1})$. The extendability problem can be decided as long as $\dim K\le 2n-3$ or $n=1,2$ or *n* is even. On the contrary, the extendability of maps into a sphere—as well as triviality of $U_0(f,r)$—cannot be decided for $\dim K\ge 2n-2$ and *n* odd, see [14].^7^ In this paper we shift our attention to higher well groups.

*Higher Well Groups—Extendability Revisited.* The main idea of our study of well groups is based on the following. We try to find *r*-perturbations of *f* with as small zero set as possible, that is, avoiding zero on $X'$ for $X'\subseteq X$ as large as possible. It is shown in [13, Lem. 3.1] that for each strict *r*-perturbation *g* of *f* we can find an extension $e:X\rightarrow {\mathbb {R}}^n$ of $f|_A$ with $g^{-1}(0)=e^{-1}(0)$ and vice versa. Thus equivalently, we try to extend $f|_A$ to a map $X'\rightarrow S^{n-1}$ for $X'$ as large as possible. The higher skeleton^8^ of *X* we cover, the more well groups we kill.

#### Observation 1

Let $f:K\rightarrow {\mathbb {R}}^n$ be a map on a compact space. Assume that the pair of spaces $A\subseteq X$ defined as $|f|^{-1}(r)\subseteq |f|^{-1}[0,r]$, respectively, can be triangulated and $\dim X=m$. If the map $f|_A$ can be extended to a map $A\cup X^ {(i-1)} \rightarrow S^{n-1}$ then $U_j(f,r)$ is trivial for $j>m-i$.

Assume, in addition, that there is no extension $A\cup X^{(i)}\rightarrow S^{n-1}$. By the connectivity of the sphere $S^{n-1}$, we have $i\ge n$. Does the lack of extendability to $X^{(i)}$ relate to higher well groups, especially $U_{m-i}(f,r)$? The answer is *yes* when $i=n$ as we show in our computability results below. On the other hand, when $i>n$, the lack of extendability *is not* necessarily reflected by $U_{m-i}(f,r)$. This leads to the incompleteness results we show in the second part of the paper.

*The Primary Obstruction.* The lack of extendability of $f|_A$ to the *n*-skeleton is measured by the so called *primary obstruction* that is defined in terms of cohomology theory as follows. We can view *f* as a map of pairs $(X,A)\rightarrow (B^n, S^{n-1})$ where $B^n$ is the ball bounded by the sphere $S^{n-1}:=\{x:|x|= r\}$. Then the primary obstruction $\phi _f$ is equal to the pullback $f^*(\xi )\in H^n(X,A)$ of the fundamental cohomology class $\xi ^n\in H^n(B^n,S^{n-1})$.^9^

#### Theorem 1

Let $$B\subseteq K$$ be compact spaces, $$f: K\rightarrow {\mathbb {R}}^n$$ be continuous, $$r>0$$, and assume the choice of *Čech (co)homology theory*. Let $$|f|^{-1}[0,r]$$ and $$ |f|^{-1}(r)$$ be denoted by *X* and *A*, respectively, and $$\phi _f$$ be the primary obstruction. Then $$\phi _f\frown H_k(X,A\cup B)$$ is a subgroup^10^ of $$U_{k-n}(f,r)$$ for each $$k\ge n$$.

After the proof of Theorem 1 we will show that the groups $\phi _f\frown H_k(X,A\cup B)$ are not only subgroups of well groups but they also fit into a natural sequence of homomorphisms for varying *r*. The resulting persistence module is then a submodule of so called well module.

Our assumptions on computer representation allow for simplicial approximation of *X*, *A* and *f*. The pullback of $\xi ^n\in H^n(B^n,S^{n-1})$ and the cap product can be computed by standard formulas. This together with more details worked out in the proof in Sect. 3 gives the following.

#### Theorem 2

Under the assumption on computer representation of *K*, *B* and *f* as above, the subgroup $\phi _f\frown H_k(X,A\cup B)$ of $U_{k-n}(f,r)$ (as in Theorem 1) can be computed. The running time is polynomial when the dimension of *K* is fixed.

*The Gap Between* $U_{k-n}$ *and* $\phi _f\frown H_k(X,A\cup B)$. There are maps *f* with $\phi _f$ trivial but nontrivial $U_0(f,r)$.^11^ But this can be detected by the above mentioned extendability criterion. We do not present an example where $U_{k-n}(f,r)\ne \phi _f\frown H_k(X,A\cup B)$ for $k-n>0$, although the inequality is possible in general. In the rest of the paper we work in the other direction to show that there is no gap in various cases and various dimensions.

An important instance of Theorem 1 is the case when *X* can be equipped with the structure of a smooth orientable manifold.

#### Theorem 3

Let $f:K\rightarrow {\mathbb {R}}^n$ and *X*, *A* be as above. Assume that *X* can be equipped with a smooth orientable manifold structure, $A=\partial X$, $B=\emptyset$ and $n+1\le m\le 2n-3$ for $m=\dim X$. Then

$$\begin{aligned} U_{m-n}(f,r)=\phi _f\frown H_m(X,\partial X). \end{aligned}$$

When $m=n$, the well group $U_0(f,r)$ can be strictly larger than $\phi _f\frown H_n(X,\partial X)$ but it can be computed.

We believe that the same claim holds when *X* is an orientable PL manifold. It remains open whether the last equation holds also for $m> 2n-3$. Throughout the proof of Theorem 3, we will show that if $g: K\rightarrow {\mathbb {R}}^n$ is a smooth *r*-perturbation of *f* transverse to 0, then the fundamental class of $g^{-1}(0)$ is mapped to the Poincaré dual of the primary obstruction. This also holds if $B\ne \emptyset$ and in all dimensions.

### Well Groups $U_*(f,r)$ are Incomplete as an Invariant of $Z_r(f)$

A simple example illustrating Theorem 3 is the map $f:S^2\times B^3\rightarrow {\mathbb {R}}^3$ defined by $f(x,y):=y$ with $B^3$ considered as the unit ball in ${\mathbb {R}}^3$. It is easy to show that

1 $$\begin{aligned}&\hbox {for every }1\hbox {-perturbation }g \hbox { of }f\hbox { and every }x\in S^2 \nonumber \\&\hbox {there is a root of }g\hbox { in }\{x\}\times B^3. \end{aligned}$$

This robust property is nicely captured by (and can be also derived from) the fact $U_2(f,1) \cong {\mathbb {Z}}$.

The main question of Sect. 4 is what happens, when the primary obstruction $\phi _f$ is trivial—and thus $f|_A$ can be extended to $X^{(n)}$—but the map $f|_A$ cannot be extended to whole of *X*. The zero set of *f* can still have various robust properties such as (1). It is the case of $f:S^2\times B^4\rightarrow {\mathbb {R}}^3$ defined by $f(x,y):=|y|{\eta }(y/|y|)$ where ${\eta }:S^3\rightarrow S^2$ is a homotopically nontrivial map such as the Hopf map. The zero set of each *r*-perturbation *g* of *f* intersects each section $\{x\}\times B_4$, but unlike in the example before, well groups do not capture this property. All well groups $U_j(f,r)$ are trivial for $j>0$ and,^12^ consequently, they cannot distinguish *f* from another $f'$ having only a single robust root in *X*. We will describe the construction of such $f'$ for a wider range examples.

In the following, $B^i_q$ will denote the *i*-dimensional ball of radius *q*, that is, $B^i_q=\{y\in {\mathbb {R}}^i:|y|\le q\}$. We also emphasize that this and the following theorem hold for arbitrary coefficient group of the homology theory $H_*$.

#### Theorem 4

Let $i,m, n\in {\mathbb {N}}$ be such that $m-i<n<i< (m+n+1) /2$ and both $\pi _{i-1} (S^{n-1})$ and $\pi _{m-1}(S^{n-1})$ are nontrivial. Then on $K=S^{m-i}\times B^i_1$ we can define two maps $f,f':K\rightarrow {\mathbb {R}}^n$ such that for all $r\in (0,1]$

- *f*, $f'$ induce the same $X= S^{m-i}\times B^i_r$ and $A=\partial X$ and have the same well groups for any coefficient group of the homology theory $H_*$ defining the well groups,
- but $$Z_r(f)\ne Z_r(f')$$.

In particular, the property

$$\begin{aligned} \hbox {for each } Z\in Z_r(.)\hbox { and } x\in S^{m-i}\ \hbox { there exists } y\in B^i_r\hbox { such that } (x,y)\in Z \end{aligned}$$

is satisfied for *f* but not for $f'$. Namely, $Z_\varepsilon (f')$ contains a singleton for each $\varepsilon >0$.

In Sect. 4 we discuss that the maps *f* and $f'$ are no peculiar examples but rather typical choices given that the underlying space *K* is the solid torus $S^{m-i}\times B^i$ and that both $Z_r(\cdot )$ are nontrivial. Further we indicate that the same result holds for even more realistic choice of the underlying space $K=B^m$ and $B=\partial K$. For the sake of exposition, we chose the case where *f* is large on the boundary of *K* and we do not need to consider nonempty *B*.

*The Lack of Extendability Not Reflected by* $U_{m-i}(f,r)$. The key property of the example of Theorem 4 is that the maps $f|_A$ and $f'|_A$ can be extended to the $(i-1)$-skeleton $X^{(i-1)}$ of *X*, for $i>n$. The difference between the maps lies in the extendability to $X^{(i)}$. Unlike in the case when $i=n$, the lack of extendability is not reflected by the well groups. The crucial part is the triviality of the well groups in dimension $m-i$ and^13^ this triviality holds in greater generality:

#### Theorem 5

Let $f:K\rightarrow {\mathbb {R}}^n$, $B\subseteq K$, $X:=|f|^{-1}[0,r]$ and $A:=|f|^{-1}\{r\}$. Assume that the pair (*X*, *A*) can be finitely triangulated.^14^ Further assume that $f|_A$ can be extended to a map $h: A\cup X^{(i-1)}\rightarrow S^{n-1}$ for some *i* such that $m-i<n<i< (m+n)/2$ for $m:=\dim X$. Then $U_{m-i}(f,r)=0$ for any coefficient group of the homology theory $H_*$.

The proof is all delegated to Appendix 3 as its core idea is already contained in the proof of Theorem 4. Note that in the setting of the last Theorem, $U_j$ is trivial for $j>m-i$ by Observation 1. One could ask the question of triviality in dimensions smaller than $m-i$ as well. Our favorite problem is the following one.

#### Problem 1

Let *f* be as in Theorem 5 and let $i=n+1$, that is, the primary obstruction is trivial. Is it true that all well groups $U_{j}(f,r)$ for $j\ge (m-n+2)/2$ are trivial?

The bound $j\ge (m-n+2)/2$ is not known to be necessary (we only know that the statement is not true for $j=1$). But passing the bound seems to bring various technical difficulties such as inapplicability of the Freudenthal suspension theorem.

*Our Subjective Conclusion on Well Groups* of ${\mathbb {R}}^n$-*Valued Maps.* We find the problem of the computability of well groups interesting and challenging with connections to homotopy theory (see also Proposition 1 below). Moreover, well groups may be accessible for non-topologists: they are based on the language of homology theory that is relatively intuitive and easy to understand. On the other hand, well groups may not have sufficient descriptive power for various situations (Theorems 4 and 5). Furthermore, despite all the effort, the computability of well groups seems far from being solved. In the following paragraph, we propose an alternative based on homotopy and obstruction theory that addresses these drawbacks.

### Related Work

*A Replacement of Well Groups of* ${\mathbb {R}}^n$-*Valued Maps.* In a companion paper [13], we find a complete invariant for an enriched version of $Z_r(f)$. The starting point is the surprising claim that $Z_r(f)$—an object of a geometric nature—is determined by terms of homotopy theory.

#### Proposition 1

([13, Thm. A]) Let $f:K\rightarrow {\mathbb {R}}^n$ be a continuous map on a compact Hausdorff domain, $r>0$, and let us denote the space $|f|^{-1}[r,\infty ]$ by $A_r$. Then the set $Z_r(f):=\{g^{-1}(0):\Vert g-f\Vert \le r\}$ is determined by the pair $(K,A_r)$ and the homotopy class of $f|_{A_r}$ in $[A_r,\{x\in {\mathbb {R}}^n:\Vert x\Vert \ge r\}]\cong [A_r,S^{n-1}]$.^15^

Note that since the well groups is a property of $Z_r(f)$, they are determined by the pair $(K,A_r)$ and the homotopy class $[f|_{A_r}]$. Thus the homotopy class has a greater descriptive power and the examples from the previous section show that this inequality is strict. If *K* is a simplicial complex, *f* is simplexwise linear and $\dim \ A_r\le 2n-4$ then $[A_r,S^{n-1}]$ has a natural structure of an Abelian group denoted by $\pi ^{n-1}(A_r).$ The restriction $\dim A_r\le 2n-4$ does not apply when $n=1,2$ and^16^ otherwise we could replace $[A_r,S^{n-1}]$ with $[A_r^{(2n-4)}, S^{n-1}]$ which contains less information but is computable. The isomorphism type of $\pi ^{n-1}(A_r)$ together with the distinguished element $[f|_{A_r}]$ can be computed essentially by [6, Thm.  1.1]. Moreover, the inclusions $A_s\subseteq A_r$ for $s\ge r$ induce computable homomorphisms between the corresponding pointed Abelian groups. Thus for a given *f* we obtain a sequence of pointed Abelian groups $\pi ^{n-1}(A_r)$ for ${r>0}$. On such sequences, there is a natural distance function (called the *interleaving distance* 17) and it can be easily shown that the distance between the sequences corresponding to a given functions *f* and $f'$ is bounded by $\Vert f-f'\Vert$. Thus after tensoring the groups by an arbitrary field, we get persistence diagrams (with a distinguished bar) that will be stable with respect to the bottleneck distance and the $L_\infty$ norm. The construction is detailed in [13].

The computation of the cohomotopy group $\pi ^{n-1}(A)$ is naturally segmented into a hierarchy of approximations of growing computational complexity. Therefore our proposal allows for a compromise between the running time and the descriptive power of the outcome. The first level of this hierarchy is the primary obstruction $\phi _f$. One could form similar modules of cohomology groups with a distinguished element as we did with the cohomotopy groups above. However, in this paper we passed to homology via cap product in order to relate it to the established well groups. In the “generic” case when *X* is a manifold no information is lost as from the Poincaré dual $\phi _f \frown [X]$ we can reconstruct the primary obstruction $\phi _f$ back.

*The Cap-Image Groups.* The groups $\phi _f\frown H_k(X,A)$ (with $B=\emptyset$) has been studied by Amit K. Patel and R. MacPherson under the name *cap-image groups.* In fact, his setting is slightly more complex with ${\mathbb {R}}^n$ replaced by arbitrary manifold *Y*. Instead of the zero sets, he considers preimages of all points of *Y* simultaneously in some sense. Although his ideas have not been published yet, they influenced our research; the application of the cap product in the context of well groups should be attributed to Patel.^18^

The advantage of the primary obstructions and the cap image groups is their computability and well understood mathematical structure. However, the incompleteness results of this paper apply to these invariants as well.

*Verification of Zeros.* An important topic in the interval computation community is the verification of the (non)existence of zeros of a given function [27]. While the nonexistence can be often verified by interval arithmetic alone, a proof of existence requires additional methods which often include topological considerations. In the case of continuous maps $f: B^n\rightarrow {\mathbb {R}}^n$, Miranda’s or Borsuk’s theorem can be used for zero verification [2, 16], or the computation of the topological degree [9, 15, 21]. Fulfilled assumptions of these tests not only yield a zero in $B^n$ but also a “robust” zero and a nontrivial 0th well group $U_0(f,r)$ for some $r>0$. Recently, topological degree has been used for simplification of vector fields [29].

The primary obstruction $\phi _f$ is the analog of the degree for underdetermined systems, that is, when $\dim K>n$ in our setting. To the best of our knowledge, this tool has not been algorithmically utilized.

## Topological Preliminaries

In this section we introduce some definitions from algebraic topology that we need throughout the proofs. The details can be found in standard textbooks, such as [18, 30].

*Simplicial Complexes and Cell Complexes.* A *simplicial complex* is a collection of simplices such that the intersection of any two of them is again a (possibly empty) simplex in the collection. It can easily be topologized and the underlying space is compact whenever the simplicial complex consists of finitely many simplices. The *i*th skeleton $X^{(i)}$ of a simplicial complex *X* is the subcomplex consisting of all simplices of dimension at most *i*. A *simplicial map* $X\rightarrow Y$ is a function that maps simplices of *X* to simplices of *Y*. It again has a simple representation as a continuous map between the underlying topological spaces.

A *cell complex* is an inductive description of a topological space *X* that consists of skeleta $X^{(0)}\subseteq X^{(1)}\subseteq \cdots \subseteq X$. The 0-skeleton $X^{(0)}$ is a discrete set and the *n*-skeleton $X^{(n)}$ is defined to be the quotient space $X^{(n-1)}\sqcup _\alpha B_\alpha ^n/\sim$ where $\{B_\alpha ^n\}$ is a collection of closed *n*-balls and the equivalence $\sim$ identifies points in $\partial B_\alpha ^n$ with points in $X^{(n-1)}$ via continuous *attaching maps* $\varphi _\alpha : S^{n-1}\rightarrow X^{(n-1)}$. The balls $B_\alpha ^n$ are called *cells* and the composition $B_\alpha ^n\hookrightarrow X^{(n-1)}\sqcup _\alpha B_\alpha ^n\rightarrow X^{(n-1)}\sqcup _\alpha B_\alpha ^n/\sim$ is called the *characteristic map* of the cell $B_\alpha ^n$. All cell complexes occurring in this paper consists of finitely many cells in which case this uniquely describes a topology of $X=\bigcup _i X^{(i)}$.

*Basic Operation on Spaces.* For pointed topological spaces (*X*, *x*) and (*Y*, *y*), the *wedge sum* $(X,x)\vee (Y,y)$ is the space $(X\sqcup Y)/\{x,y\}$, that is, *x* and *y* are identified to a single point. If the choice of the base points *x*, *y* is not important, we will simply write $X\vee Y$. The *smash product* $X\wedge Y$ is defined to be the quotient space $(X\times Y)/(X\vee Y)$. For pointed spheres $S^i$ and $S^j$, their smash product $S^i\wedge S^j$ is homeomorphic to the pointed sphere $S^{i+j}$.

For a space *X* we define the *cone* over *X* to be the space $CX:=(X\times [0,1])/(X\times \{1\})$ and the *suspension* ${\varSigma } X$ to be $CX/(X\times \{0\})$. For spheres, the identity ${\varSigma } S^i\simeq S^{i+1}$ holds. The suspension of $f: X\!\rightarrow \! Y$ is the map ${\varSigma } f: {\varSigma } X\!\rightarrow \!{\varSigma } Y$ defined by $(x,t)\mapsto [(f(x),t)]$ for $t\in [0,1]$, which converts ${\varSigma }$ into a functor $\mathrm {Top}\rightarrow \mathrm {Top}$.

*Homotopy Groups of Spheres.* For a pointed topological space (*X*, *x*), the *i* *th homotopy group of* *X* is a group which underlying space is the set of homotopy classes of maps $(S^i,*)\rightarrow (X,x)$ and the product is defined as follows. We realize the *i*-sphere as $(S^i,*)=(I^i/\partial I^i, \partial I^i/\partial I^i)$, the quotient of the unit cube $I^i$, and define $(f g)(s_1,\ldots , s_n)$ to be $f(2s_1,s_2,\ldots , s_n)$ for $s_1\in [0,1/2]$ and $g(2s_1-1,s_2,\ldots , s_n)$ for $s_1\in [1/2,1]$. This boils down to a group operation on homotopy classes of maps that is commutative whenever $i>1$. This group is denoted by $\pi _i(X,x)$. If the choice of base point *x* is not important, we will use the simpler notation $\pi _i(X)$.

Throughout this paper, we will use the facts that $\pi _i(S^i)\simeq {\mathbb {Z}}$, $\pi _3(S^2)\simeq {\mathbb {Z}}$, and that $\pi _i(S^j)$ is nontrivial for many $i>j$ (although not in all cases). The suspension operator induces a natural map ${\varSigma }: \pi _i(X)\rightarrow \pi _{i+1}({\varSigma } X)$. An important ingredient of some proofs in this paper is the *Freudenthal suspension theorem* which states that the suspension map $\pi _i(S^n)\rightarrow \pi _{i+1}(S^{n+1})$ is an isomorphism for $i<2n-1$ and a surjection for $i=2n-1$.

*(Co)Homology.* We assume that the reader is familiar with simplicial (co)homology. While we assume the input spaces *K*, *X*, *B* to be triangulable, a continuous perturbation *h* of *f* can be “wild” and have non-triangulable zero set. In its full generality, (co)homology is a (contravariant) functor from the category of topological pairs to the category of Abelian groups which satisfies Eilenberg–Steenrod axioms. These axioms are known as homotopy invariance, exactness, excision, and the dimension axiom, see [12] for exact formulations in the original source. The most common (co)homology theory is the *singular (co)homology*. We require a (co)homology theory satisfying additional axioms specified in the next section of which the Čech (co)homology is a good example. Čech homology can violate the exactness axiom in case of “wild” spaces and is therefore, strictly speaking, not a homology theory. However, it constitutes a good basis for the definition of well groups.

## Computing Lower Bounds on Well Groups

*Homology Theory Behind the Well Groups.* For computing the approximation $\phi _f\frown H_k(X,A\cup B)$ of well group $U_{k-n}(f)$ we only have to work with simplicial complexes and simplicial maps for which all homology theories satisfying the Eilenberg–Steenrod axioms are naturally equivalent. Hence, regardless of the homology theory $H_*$ used, we can do the computations in simplicial homology. Therefore the standard algorithms of computational topology [10] and the formula for the cap product of a simplicial cycle and cocycle [28, Sect.  2.2] will do the job.

The need for a carefully chosen homology theory stems from the courageous claim that the zero set *Z* of *arbitrary* continuous perturbation *supports* $\phi _f\frown \beta$ for any $\beta \in H_*(X,A\cup B)$, i.e. some element of $H_*(Z,B)$ is mapped by the inclusion-induced map to $\phi _f\frown \beta$. Without more restrictions on the perturbations, the zero sets can be “wild” non-triangulable topological spaces that can fool singular homology and render this claim false and—to the best of our knowledge—make well groups trivial. See an example after the proof of Theorem 1.

For the purpose of the work with the general zero sets, we will require that our homology theory satisfies the Eilenberg–Steenrod axioms with a possible exception of the exactness axiom, and these additional properties:

1. *Weak Continuity Property*: for an inverse sequence of compact pairs
  $$\begin{aligned} (X_0,B_0)\supset (X_1,B_1)\supset \cdots \end{aligned}$$
  the homomorphism $H_*\varprojlim (X_i,B_i)\rightarrow \varprojlim H_*(X_i,B_i)$ induced by the family of inclusion $\varprojlim (X_i,B_i)=\bigcap (X_i,B_i)\hookrightarrow (X_j,B_j)$ *is surjective.*
2. *Strong Excision*: Let $f:(X,X')\rightarrow (Y,Y')$ be a map of compact pairs that maps $X\setminus X'$ homeomorphically onto $Y\setminus Y'$. Then $f_*:H_*(X,X')\rightarrow H_*(Y,Y')$ *is an isomorphism*.

An intuitive explanation of how are the properties will be used in the proof of Theorem 1 is the following: we work with a sequence of shrinking neighborhoods $X_0, X_1,\ldots$ of the zero sets of our a given perturbation *g* of *f*. In these neighborhood we can identify the “look-for homology classes” with the help of the excision (but in the case $\Vert g-f\Vert =r$ we have to use the strong excision above). Finally, by using the weak continuity property we can identify the looked-for homology class in the infinite intersection $\bigcap _iX_i$—the zero set $g^{-1}(0)$—as well.

Čech homology theory satisfies these properties as well as the Eilenberg–Steenrod axioms with the exception of the exactness axiom, and coincides with simplicial homology for triangulable spaces [31, Chap. 6].

In addition, we need a cohomology theory $H^*$ that satisfies the Eilenberg–Steenrod axioms and is paired with $H_*$ via a cap product $H^n(X,A)\otimes H_k(X,A\cup B)\mathop {\longrightarrow }\limits ^{\frown } H_{k-n}(X,B)$ that is natural^19^ and coincides with the simplicial cap product when applied to simplicial complexes. We have not found any reference for the definition of cap product in Čech (co)homology, so we present our own construction in Appendix 4. However, if (*X*, *A*) is a triangulable pair, then we may as well use simplicial cap product and identify $\phi _f\frown H_*(X,A\cup B)$ with the corresponding subgroup of our homology theory. After slight changes in the proof of Theorem 1, all cap products could be only applied to triangulable spaces. Thus Theorem 1 would still hold under the assumption that the pair (*X*, *A*) can be triangulated, that is, the expression $\phi _f\frown H_k(X,A\cup B)$ makes sense there. At least for computability results, this is no severe restriction. With this in mind, we might as well use the Steenrod homology theory of compact metrizable spaces [24] with cap product defined simplicially on triangulable spaces. The advantage of Steenrod homology is that it satisfies the exactness axiom. We also believe that it is possible to pair it with a suitable cohomology theory by a cap product but we do not know how.

### Proof of Theorem 1

We will not explicitly deal with the definition of Čech (co)homology and will only use the facts that it satisfies the weak continuity property, strong excision, axiom of homotopy invariance, and naturality of the cap product.

We need to show that for any map *g* with $\Vert g-f\Vert \le r$, the image of the inclusion-induced map

$$\begin{aligned} H_*(g^{-1}(0), B)\rightarrow H_*(X,B) \end{aligned}$$

contains the cap product of the primary obstruction $\phi _f:=f^*(\xi )$ with all relative homology classes of $(X, A\cup B)$. Let us first restrict to the less technical case of *g* being a strict *r*-perturbation, that is, $\Vert g-f\Vert <r$.

**Fig. 2:**
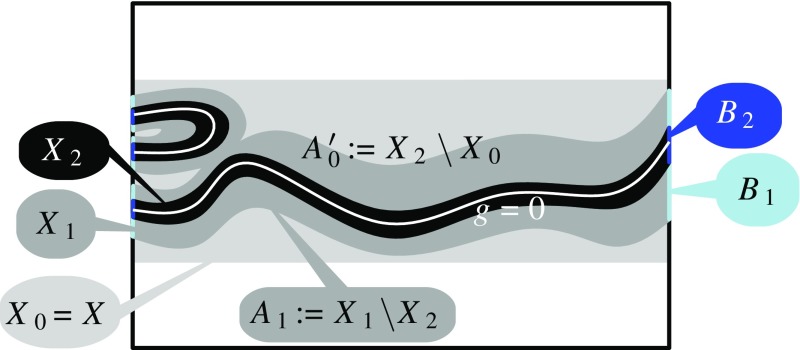
Example of spaces defined in the proof of Theorem 1

Let us denote $X_0:=X=|f|^{-1}[0,r]$ and $A_0:=A=|f|^{-1}(r)$. Next we choose a decreasing positive sequence $\varepsilon _1>\varepsilon _2>\ldots$ with $\lim _{i\rightarrow \infty } \varepsilon _i=0$ and with $\varepsilon _1<r-\Vert f-g\Vert .$ Then we for each $i>0$ we define

- $$X_i:= |g |^{-1}[0, \varepsilon _i]$$,
- $$A_{i}:=X_{i}\setminus X_{i+1}$$,
- $$A'_{i-1}:=X_{i-1}\setminus X_{i+1}$$, and,
- $$B_{i-1}:=B\cap X_{i-1}$$.

See Fig. 2 for an illustration of the spaces defined above. Note that $\bigcap _i X_i=g^{-1}(0)$, and consequently, $\bigcap _i B_i=g^{-1}(0)\cap B$. For any given $\beta \in H_k(X,A\cup B)$, our strategy is to find homology classes $\alpha _i\in H_{k-n}(X_i,B_i),$ with $\alpha _0=\phi _f\frown \beta$, that fit into the sequence of maps $H_{k-n}(X_0,B_0) \leftarrow H_{k-n}(X_1,B_1)\leftarrow \ldots$ induced by inclusions. This gives an element in $\varprojlim H_{k-n}(X_i,B_i)$, and consequently, by the weak continuity property (requirement 1 above), we get the desired element $\alpha \in H_{k-n}(g^{-1}(0),B)$.

The elements $\alpha _i$ will be constructed as cap products. To that end, we need to obtain “analogs” of $\beta$ and for that we will need a more complicated sequence of maps. It is the zig–zag sequence

2 $$\begin{aligned} X_0\mathop {\rightarrow }\limits ^{{{\mathrm{id}}}}X_0\mathop {\hookleftarrow }\limits ^{{{\mathrm{incl}}}}X_1 \mathop {\rightarrow }\limits ^{{{\mathrm{id}}}} X_1 \mathop {\hookleftarrow }\limits ^{{{\mathrm{incl}}}} X_2 \mathop {\rightarrow }\limits ^{{{\mathrm{id}}}}\cdots \end{aligned}$$

that restricts to the zig–zags

3 $$\begin{aligned} A_0\mathop {\hookrightarrow }\limits ^{{{\mathrm{incl}}}}A'_0\mathop { \hookleftarrow }\limits ^{{{\mathrm{incl}}}}A_1 \mathop {\hookrightarrow }\limits ^{{{\mathrm{incl}}}} A'_1\mathop {\hookleftarrow }\limits ^{{{\mathrm{incl}}}} A_2 \mathop {\hookrightarrow }\limits ^{{{\mathrm{incl}}}}\cdots \end{aligned}$$

and

4 $$\begin{aligned} A_0\cup B_0\mathop {\hookrightarrow }\limits ^{{{\mathrm{incl}}}}A'_0\cup B_0\mathop {\hookleftarrow }\limits ^{{{\mathrm{incl}}}}A_1\cup B_1 \mathop {\hookrightarrow }\limits ^{{{\mathrm{incl}}}} A'_1\cup B_1\mathop {\hookleftarrow }\limits ^{{{\mathrm{incl}}}} A_2\cup B_2 \mathop {\hookrightarrow }\limits ^{{{\mathrm{incl}}}}\cdots \end{aligned}$$

The pair $(X_{i+1},A_{i+1}\cup B_{i+1})$ is obtained from $(X_i,A'_i\cup B_{i})$ by excision of $|g|^{-1}(\varepsilon _{i+1},\varepsilon _i]$, that is, $X_{i+1}=X_i\setminus |g|^{-1}(\varepsilon _{i+1},\varepsilon _i]$ and $A_{i+1}\cup B_{i+1}=(A'_i\cup B_i)\setminus |g|^{-1}(\varepsilon _{i+1},\varepsilon _i]$. Hence by excision,^20^ each inclusion of the pairs $(X_i,A'_i\cup B_i)\hookrightarrow (X_{i+1},A_{i+1}\cup B_{i+1})$ induces isomorphism on relative homology groups. Therefore the zig–zag sequences (2) and (4) induce a sequencethat can be made pointed by choosing the distinguished homology classes $\beta _i\in H_k(X_i,A_i\cup B_i)$ and $\beta '_i\in H_k(X_i,A'_i\cup B_i)$ that are the images of $\beta _0:=\beta \in H_k(X_,A\cup B)$ in this sequence.

Similarly, we want to construct a pointed zig–zag sequence in cohomology induced by (2) and (3). The distinguished elements $\phi _i\in H^n(X_i,A_i)$ and $\phi '_i\in H^n(X_i,A'_i)$ are defined as the pullbacks of the fundamental cohomology class $\xi \in H^n({\mathbb {R}}^n,{\mathbb {R}}^n\setminus \{0\})$ by the restrictions of *g*. Because of the functoriality of cohomology, $\phi _i$ and $\phi _i'$ fit into the sequence induced by (2) and (3):Since *g* is an *r*-perturbation of *f* and thus $g|_{(X,A)}$ is homotopic to $f|_{(X,A)}$ via the straight line homotopy, we have that $\phi _0=\phi _f\in H^n(X,A)$.

From the naturality of the cap product we get that the elements $\phi _i\frown \beta _i$ and $\phi _i'\frown \beta '_i$ fit into the sequencethat is induced by (2), that is, each $H_{k-n} (X_i,B_i) \mathop {\cong }\limits ^{{{\mathrm{id}}}} H_{k-n}(X_i,B_i)$ is induced by the identity $X_i\mathop {\rightarrow }\limits ^{\cong } X_i$ and each map $H_{k-n}(X_i,B_i) \leftarrow H_{k-n}(X_{i+1}, B_{i+1})$ is induced by the inclusion $X_i\hookleftarrow X_{i+1}$. Hence $\alpha _i:=\phi _i\frown \beta _i$ are the desired elements and thus there is an element ${{\tilde{\alpha }}}:=(\alpha _0,\alpha _1,\ldots )$ in $\varprojlim H_{k-n}(X_i,B_i)$.

We recall that the weak continuity property of the homology theory $H_*$ assures the surjectivity of the the map

5 $$\begin{aligned} (\iota _i)_{i\ge 0}:H_{k-n}\big (\bigcap X_i,B\big )\rightarrow \varprojlim H_{k-n}(X_i,B), \end{aligned}$$

where each component $\iota _i$ is induced by the inclusion $\bigcap _i X_i\hookrightarrow X_i$. Let $\alpha \in H_{k-n}(g^{-1}(0), B)$ be arbitrary preimage of ${{\tilde{\alpha }}}$ under the surjection (5). By construction, $\alpha$ is mapped to $\alpha _0=\phi _f\frown \beta$ by the map $\iota _0$.

It remains to prove the theorem in the case when $\Vert g-f\Vert =r$. The proof goes along the same lines with only the following differences:

- For arbitrary decreasing sequence $1=\varepsilon _0>\varepsilon _1>\varepsilon _2> \cdots$ with $\lim \varepsilon _i=0$ we define $h_i:=\varepsilon _i f+ (1-\varepsilon _i)g$ for $i\ge 0$. We will furthermore need that $2\varepsilon _{i+1}>\varepsilon _i$ for every $i\ge 0$. Let  We have $A_i\subseteq A'_i$ because by definition $\Vert h_i-h_{i+1}\Vert \le (\varepsilon _i -\varepsilon _{i+1})r$ and thus $|h_i(x)|= \varepsilon _i r$ implies $|h_{i+1}(x)|\ge \varepsilon _{i+1} r$. Similarly $A_{i+1}\subseteq A'_i$ and $X_{i+1}\subseteq X_i$. Therefore as before, the zig–zag sequence (2) restricts to (3) and (4).
- The homology classes $\beta _i$ and $\beta '_i$ are defined as above. We only need to use the strong excision for the inclusion $(X_i,A'_i\cup B_i)\hookleftarrow (X_{i+1},A_{i+1}\cup B_{i+1})$.
- We define the cohomology classes $\phi _i:=h_i^*(\xi )$ and $\phi _i':= h_{i+1}^*(\xi )$. We only need to check that $h_i$ is homotopic to $h_{i+1}$ as a map of pairs $(X_i,A'_i)\rightarrow ({\mathbb {R}}^n,{\mathbb {R}}^n\setminus \{0\})$. Indeed, they are homotopic via the straight-line homotopy since $|h_{i+1}(x)|\ge \varepsilon _{i+1}r$ implies $|h_i(x)|\ge \varepsilon _ {i+1}r- (\varepsilon _i-\varepsilon _{i+1})r=(2\varepsilon _{i+1}-\varepsilon _i)r >0$. We used the inequality $2\varepsilon _{i+1}>\varepsilon _i$ which was our requirement on the sequence $(\varepsilon _i)_{i>0}$. We also have $\phi _0=\phi _f$ as $h_0=f$ and $(X_0,A_0)=(X,A)$.
- We continue by defining cap products $\alpha _i$, their limit ${{\tilde{\alpha }}}$ and its preimage $\alpha$ under the surjection $H_{k-n}(\bigcap _i X_i,B)\rightarrow \varprojlim _i H_{k-n}(X_i,B)$. To finish the proof we claim that $\bigcap _i X_i=g^{-1}(0)$. Indeed, $g(x)=0$ implies $h_i(x)\le \Vert h_i-g\Vert =\varepsilon _i r$ for each *i* and $g(x)>0$ implies $h_i(x)>0$ for *i* such that $2\varepsilon _i r<|g(x)|$.

$$\square $$

*Importance of the Choice of Homology Theory.* The surjectivity of (5) and the strong excision is not only a crucial step for Theorem 1 but implicitly also for the results stated in [4, p. 16]. If we defined well groups by means of singular homology, then even in a basic example $f(x,y)=x^2+y^2-2$ and $r=1$, the first well group $U_1(f,r)$ would be trivial. The zero set of any 1-perturbation *g* is contained in the annulus $X:=\{(x,y):\,\,1 \le x^2+y^2\le 3\}$ and the two components of $\partial X$ are not in the same connected components of $\{x\in X:\,g(x)\ne 0\}$. However, we could construct a “wild” 1-perturbation *g* of *f* such that $g^{-1}(0)$ is a Warsaw circle [23] which is, roughly speaking, a circle with infinite length, trivial first singular homology, but nontrivial Čech homology. Thus Čech homology serves as a better theoretical basis for the well groups. Another solution to avoid problems with wild zero sets would be to restrict ourselves to “nice” perturbations, for example piecewise linear or smooth and transverse to 0. Such approach would lead, to the best of our knowledge, to identical results.

### Proof of Theorem 2

Assume that the dimension of *K* is fixed. Under the assumption on computer representation of *K* and *f*, the pair (*X*, *A*) is homeomorphic to a polynomial-time computable simplicial pair $(X',A')$ such that $X'$ is a subcomplex of a subdivision $K'$ of *K* [14, Lem. 3.4]. Therefore, the induced triangulation $B'$ of $B\cap X'$ is a subcomplex of $X'$. Furthermore, a simplicial approximation $f' :A'\rightarrow S'$ of $f|_A:A\rightarrow S^{n-1}$ can be computed in polynomial time. The computation is implicit in the proof of Theorem 1.2 in [14] where the sphere $S^{n-1}$ is approximated by the boundary $S'$ of the *n*-dimensional cross polytope $B'$. The simplicial approximation $(X',A')\rightarrow (B',S')$ of $f|_X$ can be constructed consequently by sending each vertex of $X'\setminus A'$ to an arbitrary point in the interior of the cross polytope, say $0\in {\mathbb {R}}^n$. The pullback of a cohomology class can be computed by the definition of the induced map in simplicial cohomology. Namely, if a generator $\xi$ of $H^n(B',S')$ is represented by a cocycle that assigns 1 to a distinguished *n*-simplex $\sigma ^n\in B'$ and 0 to any other simplex, then $f^*(\xi )$ is represented by a cocycle that assigns to a simplex $\tau ^n\in X'$ the number $\pm 1$ iff $f'(\tau ^n)=\pm \sigma ^n$ and 0 otherwise.^21^ Therefore $\phi _f$ and $H_*(X,B)$ can be computed and the explicit formula for the cap product in [28, Sect. 2.1] yields the computation of $\phi _f\frown H_*(X,B)$. $\square$

*Modules and Diagrams Associated with* $\phi \frown H_*(X,A\cup B)$. If the parameter *r* varies, well groups *U*(*f*, *r*) naturally fit into a zig–zag sequence of homomorphisms called *well module* that can be converted into a *well diagram*, a multiset of real numbers indicating the death of homology classes of $X_r$ supported by all $Z\in Z_r(f)$ as *r* increases [11]. In this section, we show that the subgroups $V:=\phi \frown H_*(X,A\cup B)$ are not only subgroups of well groups for fixed *r* but they naturally form a persistence module which is a sub-module of the well module in some sense. It can further be converted to a sub-diagram of the well diagram. Unlike for well groups, the persistence module $\{V_{r}\rightarrow V_s\}_{0<s<r}$ is computable. It contains incomplete but often nontrivial information about homology classes of zero sets and their robustness.

Let $r_1>r_2>0$ and let $X_1$, $X_2$, $A_1$, $A_2$ be $|f|^{-1}[0,r_1]$, $|f|^{-1}[0,r_2]$, $|f|^{-1}\{r_1\}$, $|f|^{-1}\{r_2\}$ respectively, $\phi _1$, $\phi _2$ be the respective obstructions. Further, let $A_1':=|f|^{-1}[r_2,r_1]$ and $\phi _1'=f^*(\xi )\in H^n(X_1, A_1')$ be the pullback of the fundamental class $\xi \in H^n({\mathbb {R}}^n, {\mathbb {R}}^n\setminus \{0\})$. The inclusions $(X_1, A_1)\subseteq (X_1, A_1')\supseteq (X_2, A_2)$ induce cohomology maps that take $\phi _1'$ to $\phi _1$ resp. $\phi _2$. Let us denote, for simplicity, by $V_1$ the group $\phi _1\frown H_*(X_1, A_1\cup B)$, $V_2:=\phi _2\frown H_*(X_2, A_2\cup B)$ and $V_1':=\phi _1'\frown H_*(X_1, A_1'\cup B)$. Further, let $U_1$ resp. $U_2$ be the well groups $U(f,r_1)$ resp. $U(f, r_2)$.In this section, we analyze the relation between $V_1$ and $V_2$. First let $i_1$ be a map from $V_1$ to $V_1'$ that maps $\phi _1\frown \beta _1$ to $\phi _1'\frown i_*(\beta _1)$. By the naturality of cap product, $\phi _1\frown \beta _1=\phi _1'\frown i_*(\beta _1)$, so $i_1$ is an inclusion. By excision, there is an inclusion-induced isomorphisms $i_1': H_*(X_2, A_2\cup B)\mathop {\rightarrow }\limits ^{\sim } H_*(X_1, A_1'\cup B)$ and its inverse induces an isomorphism $i_2: V_1' \mathop {\rightarrow }\limits ^{\sim } V_2$ by mapping $\phi _1'\frown \beta _1'$ to $\phi _2\frown (i_1')^{-1}(\beta _1')$. The composition $i_2\circ i_1=:\iota _{12}$ is a homomorphism from $V_1$ to $V_2$. Being the composition of an inclusion and an isomorphism, $\iota _{12}$ is an injection and one easily verifies that the inclusion-induced map $i_{21}: H_*(X_2, B){\rightarrow } H_*(X_1, B)$ satisfies $i_{21}\circ \iota _{12}=\mathrm {id}|_{V_1}$. It follows that $\{V(r_i), \iota _{i,i+1}\}_{r_i>r_{i+1}}$ is a persistence module consisting of shrinking abelian groups and injections $V_{i}\rightarrow V_{i+1}$ for $r_i>r_{i+1}$. The relation between $\iota$ and well diagrams described in [11] is reflected by the commutative diagram above.

The rank of *U*(*r*) resp. *V*(*r*) can only decrease with increasing *r*. In [11], authors encode the properties of well groups to a well diagram that consists of pairs $\{(r_j, \mu _j)\}$ where $r_j$ is a number in which the rank of *U* decreases by $\mu _j\in {\mathbb {N}}$. Using computable information about $\{V(r)\}$, we may define a diagram consisting of pairs $(r_j', \mu _j')$ where the rank of *V*(*r*) decreases in $r_j'$ by $\mu _j'$. This is a subdiagram of the well diagram in the following sense: each $r_k'$ is then contained in $\{r_j\}_j$ and $\mu _k'\le \mu _k$.

*The Idea Behind the Proof of Theorem* 3. In the special case when *X* is a smooth *m*-manifold with $A=\partial X$, the zero set of any smooth *r*-perturbation *g* transverse to 0 is an $(m-n)$-dimensional smooth submanifold of *X*. It is not so difficult to show that its fundamental class $[g^{-1}(0)]$ is mapped by the inclusion-induced map to $\phi _f\frown [X]$, where $[X]\in H_m(X,\partial X)$ is the fundamental class of *X*. If $g^{-1}(0)$ is connected, then $H_{m-n}(g^{-1}(0))$ is generated by its fundamental class and we immediately obtain the reverse inclusion $\phi _f\frown H_m (X,A)\supseteq U_{m-n}(f,r)$. The nontrivial part in the proof of Theorem 3 is to show that in the indicated dimension range, we can find a perturbation *g* so that $g^{-1}(0)$ is connected. The full proof is in Appendix 2.

## Incompleteness of Well Groups

In this section, we study the case when the primary obstruction $\phi _f$ is trivial and thus the map $f|_A$ can be extended to a map $f^{(n)}:X^{(n)}\rightarrow S^{n-1}$ on the *n*-skeleton $X^{(n)}$ of *X*. Observation 1 (proved in Appendix 3) implies that the only possibly nontrivial well groups are $U_j(f,r)$ for $j\le m-n-1$.

The following lemma summarizes the necessary tools for the constructions of this section. They directly follow from [13, Lem.  3.1] and from [14, Lem. 3.3].

### Lemma 1

Let $f:K\rightarrow {\mathbb {R}}^n$ be a map on a compact Hausdorff space, $r>0$, and let us denote the pair of spaces $|f|^{-1}[0,r]$ and $|f|^{-1}\{r\}$ by *X* and *A*, respectively. Then

1. for each extension $e:X\rightarrow {\mathbb {R}}^n$ of $f|_A$ we can find a strict *r*-perturbation *g* of *f* with $g^{-1}(0)= e^{-1}(0)$;
2. for each *r*-perturbation *g* of *f* without a root there is an extension $e:X\rightarrow {\mathbb {R}}^n\setminus \{0\}$ of $f|_A$ (without a root).

In the following we want to show that well groups can fail to distinguish between maps with intrinsically different families of zero sets. Namely, in the following examples we present maps *f* and $f'$ with $U_0(f,r)=U_0(f',r)={\mathbb {Z}}$ for each $r\le 1$ and $U_i(f,r)=U_i(f,r)=0$ for each $r\le 1$ and $i>0$. However, $Z_r(f)$ will be significantly different from $Z_r(f')$.

### Proof of Theorem 4

We have that $B=\emptyset$ and $K=S^j\times B^i$, where $B^i$ is represented by the unit ball in ${\mathbb {R}}^i$ and $j=m-i$. Let the maps $f,f': K\rightarrow {\mathbb {R}}^n$ be defined by

$$\begin{aligned} f(x,y):=|y| \,\varphi (x,y/|y|) \quad \hbox { and }\quad f'(x,y):=|y|\varphi ' (x,y/|y|), \end{aligned}$$

where $\varphi ,\varphi ':S^j\times S^{i-1}\rightarrow S^{n-1}\subseteq {\mathbb {R}}^n$ are defined by

- $\varphi (x,y):=\mu (y)$ where $\mu : S^{i-1}\rightarrow S^{n-1}$ is an arbitrary nontrivial map.
- $\varphi '$ is defined as the composition $S^j\times S^{i-1}\rightarrow S^{m-1}\mathop {\rightarrow }\limits ^{\nu } S^{n-1}$ where the first map is the quotient map $S^j\times S^{i-1}\rightarrow S^j\wedge S^{i-1}\cong S^{m-1}$ and $\nu$ is an arbitrary nontrivial map. In other words, we require that the composition $\varphi '{\varPhi }$—where ${\varPhi }$ denotes the characteristic map of the $(m-1)$-cell of $S^j\times S^{i-1}$—is equal to the composition $\nu q$, where *q* is the quotient map $B^{m-1}\rightarrow B^{m-1}/(\partial B^{m-1})\cong S^{m-1}$.

*Well Groups Computation.* Next we prove that the well groups of $U_*(f,r)$ and $U_*(f',r)$ are the same for $r\in (0,1]$, namely, nonzero only in dimension 0, where they are isomorphic to ${\mathbb {Z}}$. We obviously have $X=S^j\times \{y\in {\mathbb {R}}^i:|y|\le r\}\simeq S^j\times B^i$ and $A=\partial X$ for both maps. The restriction $f|_A$ and $f'|_A$ are equal to $\varphi$ and $\varphi '$ (after normalization). We first prove that $U_0(f,1)\cong U_0(f',1)\cong {\mathbb {Z}}$. This fact follows from $H_0(X)\cong {\mathbb {Z}}$, from non-extendability of $\varphi$ and $\varphi '$ and from Lemma 1 part 2 (or [14, Lem. 3.3]).

### Lemma 2

The map $\varphi '$ cannot be extended to a map $X\rightarrow S^{n-1}$.

We postpone the proof to Appendix 1. Since the map $\mu :S^{i-1}\rightarrow S^{n-1}$ cannot be extended to $B^i\supset S^{i-1}$, also $\varphi$ cannot be extended to *X*.

Since then only the *j*th homology group of *X* is nontrivial, the remaining task is to show that $U_j(f,1)\cong U_j(f',1) \cong 0$. We do so by presenting two *r*-perturbations *g* and $g'$ of *f* and $f'$, respectively:

- $g(x,y):=f(x,y)-rx=|y|\mu (y/|y|)-rx$ where we consider $S^j\subseteq {\mathbb {R}}^{j+1}$ as a subset of ${\mathbb {R}}^n$ naturally embedded in the first $j+1$ coordinates (here we need that $j=m-i<n$).
- We first construct an extension $e':X\rightarrow {\mathbb {R}}^n$ of $\varphi ' =f'|_A$ and then the *r*-perturbation $g'$ is obtained by Lemma 1 part 1. The extension $e'$ is defined as constant on the single *i*-cell of *X*, that is, $e'(x_0,y)$ is put equal to the basepoint of $S^{n-1}\subseteq {\mathbb {R}}^n$. On the remaining *m*-cell $B^m\cong \{z\in {\mathbb {R}}^m:|z|\le 1\}$ of *X* we define $e'(z):=|z|e'(z/|z|)$, where each point *z* is identified with a point of *X* via the characteristic map ${\varPsi }_1:B^m\rightarrow X$ of the *m*-cell $B^m$.^22^

By definition the only root of $g'$ is the single point ${\varPsi }_1(0)$ of the interior of *X*. Therefore $U_j(f,1)\cong 0$. Note that the role of ${\varPsi }_1(0)$ could be played by an arbitrary point in the interior of *X*.^23^

The zero set $g^{-1}(0)=\{(x,y):\,|y|=r$ and $\mu (y/|y|)=x\}$ is by definition homeomorphic to the pullback (i.e., a limit) of the diagram6where $\iota$ is the equatorial embedding, i.e., sends each element *x* to $(x,0,0,\ldots )$. In plain words, the zero set is the $\mu$-preimage of the equatorial *j*-subsphere of $S^{n-1}$. We will prove that under our assumptions on dimensions, this is the $(m-n)$-sphere $S^{m-n}$. Then from $m-n>m-i=j$ it will follow that $H_j(g^{-1}(0))\cong 0$ which proves Theorem 4.

The topology of the pullback is particularly easy to see in the case when $j=n-1$ and $\iota$ is the identity. There it is simply the domain of $\mu$, that is, $S^{i-1}$ where $i-1=m-j-1=m-n$.

In the general case, the only additional tool we use to identify the pullback is the Freudenthal suspension theorem. The pullback is homeomorphic to the $\mu$-preimage of the equatorial subsphere $S^{m-i}\subseteq S^{n-1}$. By Freudenthal suspension theorem $\mu$ is homotopic to an iterated suspension ${\varSigma }^a\eta$ for some $\eta :S^{i-1-a}\rightarrow S^{n-1-a}$ assuming $i-1-a\le 2(n-1-a)-1$. We want to choose *a* so that $n-1-a=m-i$ and thus images $\mathrm{Im}(\eta )=S^{n-1-a}$ and $\mathrm{Im}(\iota )=S^j\subseteq S^{n-1}$ coincide (since $j=m-i$ by definition). The last inequality with the choice $a=n-1-m+i$ is equivalent to the bound $i\le (m+n-1)/2$ from the hypotheses of the theorem. In our example, we may have chosen *f* in such a way that $\mu ={\varSigma }^a\eta$. But even for the choices of $\mu$ only homotopic to ${\varSigma }^a\eta$ we could have changed *f* on a neighborhood of $\partial K$ by a suitable homotopy. To finish the proof we use the fact that, by the definition of suspension, the $\mu$-preimage of $S^{m-i}\subseteq S^{n-1}$ is identical to the $\eta$-preimage of $S^{m-i}$, that is $S^{i-1-j}=S^{m-n}$.

*Difference Between* $Z_r(f)$ *and* $Z_r(f')$. Because the map $\mu$ is homotopically nontrivial, the zero set of each extension $e:X\rightarrow {\mathbb {R}}^n$ of $f|_A$ intersects each “section” $\{x\}\times B^i$ of *X*. By Lemma 1 part 2 (or [14, Lem. 3.3]) applied to each restriction $f|_{\{x\}\times B^i}$, the same holds for *r*-perturbations *g* of *f* as well. In other words, the formula “for each $x\in S^j$ there is $y\in B^i$ such that $f(x,y)=0$” is *satisfied robustly*, that is

$$\begin{aligned} \forall Z\in Z_r(f):\forall x\in S^j: \exists y\in B^i: (x,y) \in Z \end{aligned}$$

is satisfied. The above formula is obviously not true for $f'$ as can be seen on the *r*-perturbations $g'$. In particular, for every $r\in (0,1]$ the family $Z_r(f')$ contains a singleton. This completes the proof of Theorem 5. $\square$

*Robust Optimization.* As an example of another relevant property of $Z_r(f)$ not captured by the well groups, we mention the following. For any given $u:K\rightarrow {\mathbb {R}}$, we may want to know what is the *r*-*robust maximum of* *u* *over the zero set of* *f*, i.e., $\inf _{Z\in Z_r(f)}\max _{z\in Z} u(z)$. Let, for instance, $u(x,y)=u(x)$ depend on the first coordinate only. Then the *r*-robust maximum for *f* is equal to $\max _{x\in S^j} u(x)$ as follows from the discussion in the previous paragraph. On the other hand, the *r*-robust maximum for $f'$ is equal to $\min _x u(x)$ and is attained in $g'$ when we set the value ${\varPsi }_1(0):=(\mathrm {arg}\,\mathrm{min}_{x\in S^j}u(x),0)$ from the proof above. This holds for *r* arbitrarily small. The robust optima constitutes another and, in our opinion, practically relevant quantity whose approximation cannot be derived from well groups.

*Further Remarks on Theorem* 4. We first want to indicate that in some sense the maps *f* and $f'$ are no peculiar examples but rather typical choices. More precisely, we assume that $r>0$ is fixed and that $X=S^j\times B^i$ and $A=\partial X$. (Note that in the natural cell structure of *X* there is only one *i*-dimensional and one $(i+j)$-dimensional cell outside of *A*.) It can be easily proved that under these assumptions the maps *f* and $f'$ can be chosen arbitrarily in such a way that

- $$f|_A$$ cannot be extended to $$X^{(i)}$$ (it extends to $$ X^{(i-1)}$$ trivially as $$A=X^{(i-1)}$$) and
- $f'|_A$ extends to $X^{(i)}$ but not to *X*.^24^

The only addition needed to prove this more general version is in the computation of $U_{m-i}(f,r)$. For that we can either use Theorem 5 when $i< (m+n)/2$ or enhance the proof of Theorem 4 when $i=(m+n)/2$ which we omit here. Note that the nonextendability properties of *f* and $f'$ require nontriviality of the homotopy groups of spheres as in the hypothesis of Theorem 4. Then only for the requirement $i>n$ we know that is strict. The other two inequalities are used to find the map $\iota$ such that the pullback (6) is connected enough. The inequality $i<(m+n+1)/2$ can be relaxed to requiring the existence of $[\mu ]\in \pi _{i-1}(S^{n-1})$ such that $[\mu ]={\varSigma }^a \eta$ for *a* sufficiently large as stated in the proof.

Finally, we remark that the same incompleteness results could be achieved for even more realistic domain $K=B^j\times B^i\cong B^m$. We only need to choose *f* and $f'$ with $X=B^j\times B^i_r$ and $A=B^j\times (\partial B^i_r)$ and with the same properties as above. Then for the natural choice $B=\partial K$ and under the same hypotheses, both well groups will be equal.

*Sketch of the Proof of Lemma* 2. The ultimate claim is that $\varphi '$ cannot be extended to the *m*-cell of *X* no matter how the extension on the *i*-cell was chosen. To this end, we need two properties of the obstruction to extendability on the *m*-cell (which is an element of $\pi _{m-1} (S^{n-1})$):

1. First, that the obstruction is independent of the choice of the extension on the *i*-cell. This essentially follows from the bilinearity of the Whitehead product $\pi _{i}(S^{n-1})\otimes \pi _{m-i}(S^{n-1})\rightarrow \pi _{m-1} (S^{n-1})$, namely, that the Whitehead product of a trivial element with an arbitrary element is again a trivial element.
2. Second, that the obstruction depends linearly on the choice of the element $[\nu ]\in \pi _{m-1}(S^{n-1})$ in the definition of the map $\varphi '$. This amounts to the basic obstruction theory and the cell structure of the solid torus.

The full proof is presented in Appendix 1.

## Figures and Tables

**Fig. 3:**
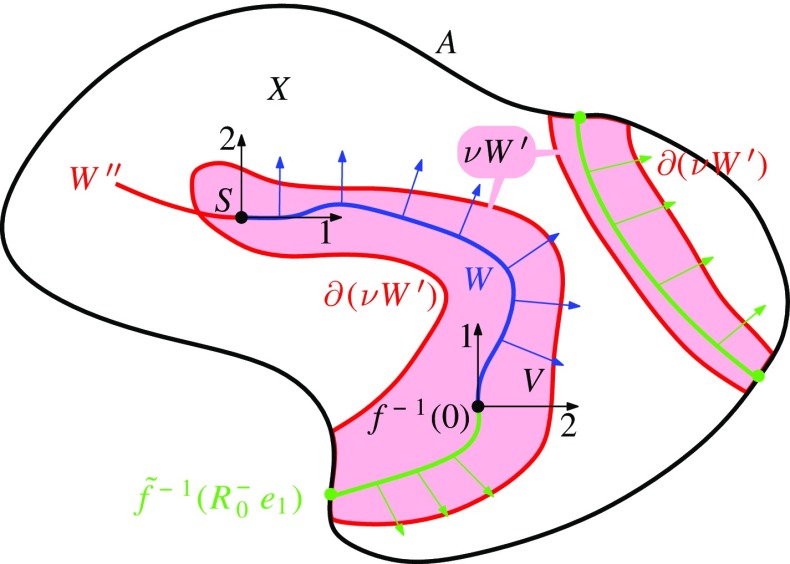
Illustration of the framed manifold $W'$ that consists of *W* and ${\tilde{f}}^{-1}{\mathbb {R}}_0^- e_1$. The boundary $\partial W'$ consists of *S* and some submanifold of $A=\partial X$

**Fig. 4:**
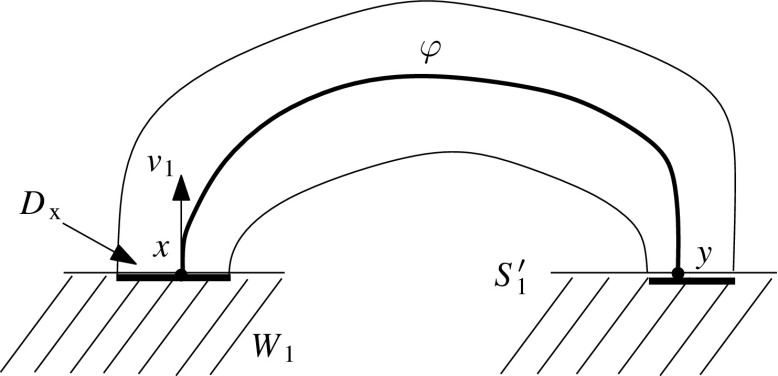
The $m-n$-dimensional neighborhoods $D_x$ of *x* and $D_y$ of *y* in $S_1'$ are connected via an $m-n+1$ dimensional tubular neighborhood *T* of a curve $\varphi$ connecting *x* and *y*

**Fig. 5:**
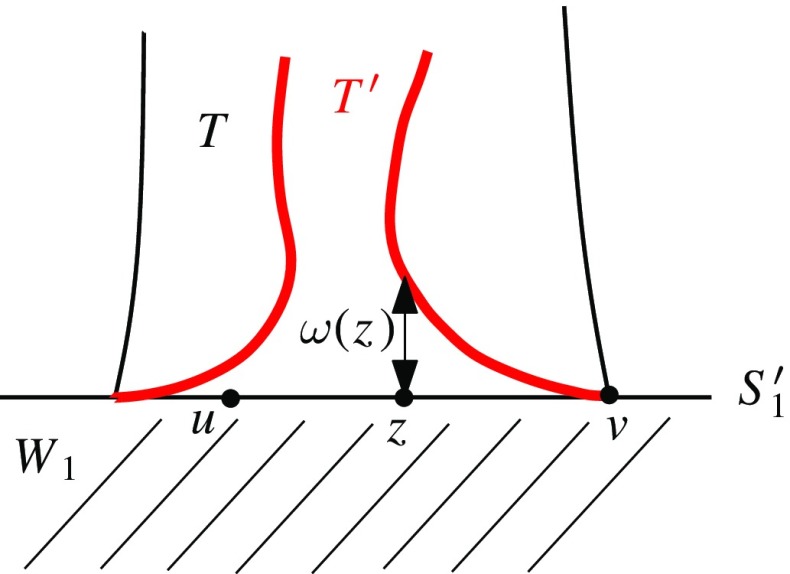
Illustration of corner smoothing. $T'\subseteq T$ is chosen so that, in a neighborhood of $v\in \partial D_x$, $T'$ consists of points in $W_1\cup T$ whose distance from $W_1$ is bounded by a function $\omega (z)$ that has zero gradient in *v*

## Appendix 1: The Nonextendability Proof (Proof of Lemma 2)

### Proof of Lemma 2

As our ultimate claim will be that $\varphi '$ cannot be extended to the *m*-cell of $X=S^j\times B^i$, we will need to analyse the gluing map of that cell. In particular, we need to establish its relation to the gluing map of the *m*-cell of $T=S^j\times S^i$. In the first row of the following commutative diagram we have the factorization of the characteristic map ${\varPsi }$ of the *m*-cell of *T* into the characteristic map ${\varPsi }_1$ of the *m*-cell of *X* and another quotient map ${\varPsi }_2$.

Note that above we identify spheres with the quotients of balls by their boundary. The restriction of each characteristic map to the boundary $\partial (B^j\times B^i)$ gives the respective gluing maps as is shown in the second row. We have that $X^{(m-1)} = \big (S^j\times \partial B^i\big ) \cup \big (\{*\}\times B^i\big )$ and^25^ indeed the quotient map ${\varPsi }_2$ (which identifies each $\{x\}\times \partial B^i$ into a point $(x,*)$) maps $X^{(m-1)}$ to $S^j\vee S^i$. The crucial consequence is that the gluing map $\psi$ of the *m*-cell in *T* is the composition of the gluing map $\psi _1$ of the *m*-cell in *X* and the restriction $\psi _2$ of the quotient map ${\varPsi }_2$ as above.

Another tool that we need is the *Whitehead product* [18, Chap. X, 7.2] for which we need the explicit construction. Again, $\psi :S^{m-1}\rightarrow S^j\vee S^{i}$ denotes the gluing map of the *m*-cell in $T=S^j\times S^{i}$. Then the homotopy class of the composition $S^{m-1}\mathop {\rightarrow }\limits ^{\psi }S^j\vee S^{i} \mathop {\longrightarrow }\limits ^{\omega _1\vee \omega _2}S^{n-1}$ defines the Whitehead product of arbitrary elements $[\omega _1]\in \pi _{j}(S^{n-1})$ and $[\omega _2]\in \pi _{i}(S^{n-1})$. We will use the bilinearity of the Whitehead product, especially, that the product of the trivial element $[{{\mathrm{const}}}]$ and arbitrary $[\omega ]$ is trivial.

Let us assume that

$$\begin{aligned} h:\underbrace{e^i\cup A}_{X^{(i)}\cup A}\rightarrow S^{n-1} \end{aligned}$$

is an extension of $\varphi '$ on the unique *i*-cell $e^i$ of *X* . The map *h* can be extended to *X* if and only if there is a nullhomotopy for the composition $h\psi _1:S^{m-1}\rightarrow S^{n-1}$ where again $\psi _1:S^{m-1}\rightarrow X^{(m-1)}=X^{(i)}\cup A$ is the gluing map of the *m*-cell of *X*. Roughly speaking, the key difficulty is that $\varphi '$ can be extended on $e^i$ in various essentially different ways (whenever $\pi _i(S^{n-1})$ is nontrivial). The key observation is that this choice does not influence the homotopy class of $h\psi$ and that it is always equal to the above chosen nontrivial element $[\nu ]\in \pi _{m-1}(S^{n-1})$ up to a sign.

Towards that end, let us form an auxiliary map $h':e^{i}\cup A\rightarrow S^{n-1}$ that is constant on $\partial X$ and equal to *h* on the unique *i*-cell of *X*. We want to show first, that $h'\psi _1$ is homotopically trivial, and second, that $[h\psi _1]=[h'\psi _1]\pm \nu$.

1. Since $h'$ is constant on $\{x\}\times (\partial B^i)$ for each $x\in S^j$, it factors through the corresponding quotient $S^j\vee S^i$ of $X^{(i)}$ as follows (in the following the factorization is preceded by $\psi _1$):
  Here ${{\mathrm{const}}}$ denotes the constant map and $\omega$ is the map determined by *h* (or equivalently by $h'$) on $\{*\}\times B^i$. Since by the above considerations $\psi _2\psi _1=\psi$, the composition $h'\psi _1$ is equal to $({{\mathrm{const}}}\!\vee \omega )\psi$—the representative of the Whitehead product of $[{{\mathrm{const}}}]$ and $[\omega ]$. By the bilinearity of the Whitehead product, $h'\psi _1$ is trivial.
2. The second claim—$$[h\psi _1]=[h'\psi _1]\pm \nu $$—follows from basic obstruction theory. This claim follows from the fact, that for any pair of maps *h* and $$h'$$ that agree on $$X^{(m-2)}$$, the difference of their obstruction cocycles $$z^m_h-z^m_{h'}\in Z^m\big (X,\pi _{m-1}(S^{n-1})\big )$$ equals the coboundary of the difference cochain $$d_{h,h'}\in C^{m-1}\big (X,\pi _{m-1}(S^{n-1} )\big )$$. To get the conclusion, we need two ingredients: that the coboundary map is an isomorphism and that the difference cochain is nontrivial, namely, that it assigns $$\nu $$ to the $$ (m-1)$$-cell of *X*. The first ingredient was already shown in the first paragraph of this proof. Since the cellular chain structure of *X* is rather simple—having one generator in both dimensions *m* and $$m-1$$—we rephrase everything in an elementary language below.
  The first ingredient is that the degree *d* of the composition
  $$\begin{aligned} S^{m-1}\mathop {\rightarrow }\limits ^{\psi _1} X^{(m-1)}\mathop {\rightarrow }\limits ^{}X^{(m-1)}/(S^j\vee B^i)\cong S^{m-1} \end{aligned}$$
  is equal to $\pm 1$. The second ingredient is that, once we denote the characteristic map of the $(m-1)$-cell of *X* by ${\varPhi }$, the *difference map* of $h'{\varPhi }$ and $h{\varPhi }$ equals $\pm \nu$. The difference map of any given maps $f:B^{m-1}\rightarrow S^{n-1}$ and $g:B^{m-1}\rightarrow S^{n-1}$ with $f|_{\partial B^{m-1}}=g|_{\partial B^{m-1}}$ is defined as $\delta _{f,g}:=f\cup _{\partial B^{m-1}} g:S^{m-1}\rightarrow S^{n-1}$. In words, *f* defines $\delta _{f,g}$ on the northern hemisphere and *g* defines $\delta _{f,g}$ on the southern hemisphere. Because there are factorizations $h{\varPhi }=\varphi '{\varPhi }=\nu q$ and^26^ $h'{\varPhi }={{\mathrm{const}}}=\nu {{\mathrm{const}}}$ through $S^{m-1}$, we have that $\delta _{h{\varPhi }, h'{\varPhi }}= \nu \delta _{q,{{\mathrm{const}}}}$. Obviously, the map $\delta _{q,{{\mathrm{const}}}}$ has degree $\pm 1$ and thus the second ingredient holds. By the definition of the addition in $\pi _{m-1}(S^{n-1})$, we have that $[h\psi _1]=[h'\psi ]\pm d[\delta _{h{\varPhi },h'{\varPhi }}]$.

This concludes the proof of Lemma 2 and thus the proof of Theorem 4 as well. $\square$

## Appendix 2: Proof of Theorem 3

*Overview.* The proof of Theorem 3 will be divided into several steps. Theorem 1 implies one inclusion and for the other, it is sufficient to find a smooth *r*-perturbation *g* of *f* such that 0 is a regular value of *g* and the homology image of $g^{-1}(0)$ in *X* generates the cap product $\phi _f\frown H_m(X,\partial X)$. First we show that the general case easily reduces to the case where *X* is connected. In the next step, we describe the $(m-n)$th homology of zero sets of perturbations transverse to zero: we prove that if 0 is a regular value of a strict *r*-perturbation *g* of *f*, then the Poincaré dual of $\phi _f$ equals the image of the fundamental class of the submanifold $g^{-1}(0)$ (Lemma 3). If $g^{-1}(0)$ is connected, then the fundamental class of $g^{-1}(0)$ generate its top homology $H_{m-n}(g^{-1}(0))$. In this way, we reduce the proof of Theorem 3 to the statement that if $n+1\le \dim X\le 2n-3$ and *X* is connected, then there exists some smooth strict *r*-perturbation *g* of *f* such that 0 is a regular value of *g* and $g^{-1}(0)$ is connected. To prove this, we introduce the notion of framed submanifolds and show that if a framed $(m-n+1)$-submanifold *W* has framed boundary consisting of $S\sqcup f^{-1}(0)$, then *S* is the zero set of some smooth strict *r*-perturbation *g* having 0 as regular value (Lemma 4). Finally, we show that there exists a framed submanifold *W* and a *connected* framed $(m-n)$-submanifold *S* of *X* s.t. $\partial W=f^{-1}(0)\sqcup S$ (Lemma 5).

*Reduction to the Case of Connected* *X*. Assume that Theorem 1 holds for *X* connected. The compact space *X* can only contain finitely many connected components, say $X_1,\ldots , X_k$. Then $H_m(X, \partial X)\simeq \sum _i H_m(X_i, \partial X_i)$ and $\phi _f\frown H_m(X, \partial X)\simeq \sum _j \iota _j^*\phi _f\frown H_m(X_j, \partial X_j)$ where $\iota _j: X_j\hookrightarrow X$ is the inclusion. If we assume that Theorem 3 holds for *X* connected, we may use it for $f|_{X_i}: X_i\rightarrow {\mathbb {R}}^n$ and get that

7 $$\begin{aligned} \bigcap _{g|_{X_i}:\,\Vert g|_{X_i}-f|_{X_i}\Vert \le r} \mathrm {Im}\big (H_{m-n}(g|_{X_i}^{-1}(0)) \mathop {\longrightarrow }\limits ^{i_*} H_{m-n}(X_i) \big ) \end{aligned}$$

is contained in $\iota _i^*\phi _f\frown H_{m}(X_i, \partial X_i)$ for all *i*. However, each *r*-perturbation *g* of *f* induces *r*-perturbations $g|_{X_i}$ of $f|_{X_i}$ and each set of *r*-perturbation $g_i$ of $f|_{X_i}$ induces an *r*-perturbation *g* of *f*; therefore

$$\begin{aligned} \bigcap _{g:\,\Vert g-f\Vert \le r} \mathrm {Im}\big (H_{m-n}(g^{-1}(0)) \mathop {\longrightarrow }\limits ^{i_*} H_{m-n}(X) \big ) \end{aligned}$$

is isomorphic to the direct sum of (7) over *i* and is therefore a subset of $\sum _j \iota _j^*\phi _f\frown H_{m}(X_j, \partial X_j)\simeq \phi _f\frown H_m(X,\partial X)$.

In the rest of the proof, we will assume that *X* is connected.

*Poincaré Dual of the Fundamental Class.* Now we will show that the Poincaré dual of the first obstruction equals the image of the fundamental class of the zero set of a smooth strict *r*-perturbation transverse to 0.

### Lemma 3

Let *X* be a smooth oriented *m*-manifold with boundary, *A* and *B* be $(m-1)$-submanifolds of $\partial X$, $\partial X=A\cup B$, $\partial A=\partial B$, $f: (X,A)\rightarrow ({\mathbb {R}}^n, {\mathbb {R}}^{n}\setminus \{0\})$ be smooth with 0 a regular value of *f* and $f|_{\partial X}$, $[X]\in H_m(X,\partial X)$ the fundamental class of *X*, $\phi _f=f^*(\xi )\in H^n(X,A)$ the first obstruction and $\phi _f\frown [X]$ its Poincaré dual.

Then the smooth submanifold $f^{-1}(0)$ of *X* can be endowed with an orientation such that its fundamental class $[f^{-1}(0)]\in H_{m-n}(f^{-1}(0), B)$ satisfies

$$\begin{aligned} i_*([f^{-1}(0)]) = \phi _f\frown [X], \end{aligned}$$

where $i: f^{-1}(0)\hookrightarrow X$ is the inclusion.

It follows immediately that $\phi _f\frown [X]$ equals to the image of the fundamental class of any smooth *g* such that $\phi _g=\phi _f$: this happens, in particular, if $A=|f|^{-1}(r)$ and *g* is a smooth strict *r*-perturbation of *f* transverse to 0.

### Proof

If 0 is a regular value of *f* and $f|_{\partial X}$, then $f^{-1}(0)$ is a smooth $(m-n)$-dimensional submanifold of *X* and $\partial f^{-1}(0)=f^{-1}(0)\cap B$ is a smooth submanifold of *B*; it also follows that the inclusion $i:f^{-1}(0)\hookrightarrow X$ induces a homology map that maps the fundamental class $[f^{-1}(0)]\in H_{m-n}(f^{-1}(0), \partial f^{-1}(0))$ to $H_{m-n}(X,B)$. Smooth manifolds can be triangulated and a triangulation of $f^{-1}(0)\cap B$ can be extended to a triangulation of all *B* and subsequently to $f^{-1}(0)$, $\partial X$ and *X* [26, Thm. 10.6.]. In the rest, we will work with simplicial (co)homology and simplicial cap product, which for simplicial complexes coincides with our homology theory. We will show that the Poincaré dual $\phi _f\frown [X]$ of the obstruction is the image of the fundamental class $[f^{-1}(0)]$ by induction on *n*.

Let $$n=1$$. For the use of simplicial homology, choose an ordering of all vertices such that the vertices in $$f^{-1}[-r, 0)$$ have lower rank then vertices in $$f^{-1}[0,r]$$. The obstruction $$\phi _f$$ can be represented by a simplicial cocycle $$z_f$$ that assigns 1 to each oriented 1-simplex *xy* with $$f(x)<0$$ and $$f(y)\ge 0$$, and 0 to other oriented 1-simplices. The fundamental class $$[X]\in H_m(X, \partial X)$$ consists of all *m*-simplices in *X* and the cap product $$z_f\frown (\sum _{{\Delta }_m\in X^{(m)}} {\Delta }_m)$$ consists of all $$(m-1)$$-simplices $${\Delta }_{m-1}=[y_0,y_1,\ldots , y_{m-1}]$$ in $$f^{-1}(0)$$ such that $$[x, y_0, \ldots , y_{m-1}]$$ is an *m*-simplex in *X*, $$f(x)<0$$ and $$f(y_j)=0$$ for all *j*. The set of all such $${\Delta }_{m-1}$$ yields a triangulation of $$f^{-1}(0)$$ with the orientation induced from the chosen orientation of *X*. It follows that $$\phi _f\frown [X]$$ equals the image of the fundamental class of $$[f^{-1}(0)]$$ in $$H_{m-1}(X,B)$$.

Let $n>1$ and $f=(f_1, f_2)$ with $f_1$ scalar valued and $f_2: X\rightarrow {\mathbb {R}}^{n-1}$. Each $x\in f^{-1}(0)$ is a regular point of *f* and $f|_{\partial X}$, hence it is a regular point of both $f_1$, $f_1|_{\partial X}$ and $f_2$, $f_2|_{\partial X}$. It follows that there exists a neighborhood *U* of $f^{-1}(0)$ s.t. 0 is a regular value of both $f_1|_U$, $f_1|_{U\cap \partial X}$ and $f_2|_U$, $f_2|_{U\cap \partial X}$. Possibly changing $f_1$ and $f_2$ outside *U* without changing $f^{-1}(0)=f_1^{-1}(0)\cap f_2^{-1}(0)$, we may assume that 0 is a regular value of both $f_1$, $f_1|_{\partial X}$ and $f_2$, $f_2|_{\partial X}$, so that $f_1^{-1}(0)$ and $f_2^{-1}(0)$ are smooth manifolds of dimensions $m-1$ and $m-n+1$, respectively. Choose a compact $(m-1)$-submanifold $A_1$ of *A* such that $0\notin f_1(A_1)$ and so that $A_2:=\overline{A\setminus A_1}$ satisfies $0\notin f_2(A_2)$. Both $A_2$ and $B\cup A_1=\overline{\partial X\setminus A_2}$ are smooth $m-1$-dimensional submanifolds of $\partial X$, $A_2\cup (B\cup A_1)=\partial X$ and $\partial A_2=\partial (B\cup A_1)$.

The maps $f, f_1$ and $f_2$ can be considered as maps of pairs $f: (X,A)\rightarrow ({\mathbb {R}}^n, {\mathbb {R}}^{n}\setminus \{0\})$, $f_1: (X, A_1)\rightarrow ({\mathbb {R}}, {\mathbb {R}}\setminus \{0\})$ and $f_2: (X, A_2): ({\mathbb {R}}^{n-1}, {\mathbb {R}}^{n-1}\setminus \{0\})$. Let $\xi ^n, \xi ^1$, resp. $\xi ^{n-1}$ be fundamental classes of $H^j({\mathbb {R}}^j, {\mathbb {R}}^j\setminus \{0\})$ where *j* equals *n*, 1, resp. $n-1$; here we assume a canonical orientation on ${\mathbb {R}}^j$. Let $\phi _1:=f_1^*(\xi ^1)\in H^1(X,A_1)$ and $\phi _2:=f_2^*(\xi ^{n-1})\in H^{n-1}(X, A_2)$ be the corresponding obstructions. The cross product in cohomology [18, p. 214]

$$\begin{aligned} H^1({\mathbb {R}}, {\mathbb {R}}\setminus \{0\})\times H^{n-1}({\mathbb {R}}^{n-1}, {\mathbb {R}}^{n-1}\setminus \{0\}) \mathop {\longrightarrow }\limits ^{\times } H^n({\mathbb {R}}^n, {\mathbb {R}}^n\setminus \{0\}) \end{aligned}$$

takes $(\xi ^1, \xi ^{n-1})$ to $\xi ^n$. Using this we obtain

$$\begin{aligned} f^*(\xi ^n)= f^*(\xi ^1\times \xi ^{n-1})=(p_1 f)^* (\xi ^1) \smile (p_2 f)^*(\xi ^{n-1})=f_1^*(\xi ^1)\smile f_2^*(\xi ^{n-1}) \end{aligned}$$

for $p_1$ and $p_2$ the projections in $B^n$ to the first, resp. the remaining components. Comparing the left and right hand side of the last equation yields $\phi _f=f^*(\xi ^n)=f_1^*(\xi _1)\smile f_2^*(\xi ^{n-1})=\phi _1\smile \phi _2$.

Now we use the induction hypothesis for the ${\mathbb {R}}^{n-1}$-valued map $f_2$ and the subcomplexes $A_2$ and $B\cup A_1$ of *X*. It says that $\phi _2\frown [X]$ is the image of the fundamental class $[f_2^{-1}(0)]\in H_{m-n+1}(f_2^{-1}(0), B\cup A_1)$ in $H_{m-n+1}(X, B\cup A_1)$.

The naturality of the cap product yields the following scheme:

8

The pullback ${\tilde{\phi }}_1:=i^*\phi _1\in H^1(f_2^{-1}(0), A_1)$ is the obstruction associated to the restriction of $f_1$ to $f_2^{-1}(0)$. The restrictions $f_1|_{f_2^{-1}(0)}$ as well as $f_1|_{\partial f_2^{-1}(0)}$ have 0 as regular value, so using again the induction hypothesis, we get that ${\tilde{\phi }}_1\frown [f_2^{-1}(0)]$ is the inclusion-induced image of $[f^{-1}(0)]\in H_{m-n}(f^{-1}(0), B)$ in $H_{m-n}(f_2^{-1}(0), B)$. Using the commutativity of diagram (8), we get that the inclusion-induced image of $[f^{-1}(0)]$ in $H_{m-n}(X,B)$ equals

$$\begin{aligned} \phi _1\frown (\phi _2\frown [X])=(\phi _1\smile \phi _2) \frown [X]=\phi _f\frown [X] \end{aligned}$$

which completes the proof. $\square$

In the rest of this section, we will only need the last lemma for the case where $A=\partial X$ and $B=\emptyset$.

*Reduction to the Existence of a Perturbation* *g* *Such That* $g^{-1}(0)$ *is Connected.* Assume that $(X,A)=|f|^{-1}[0,r], |f|^{-1}(r)$, *X* is a smooth connected manifold, $A=\partial X$, *g* is smooth, 0 is a regular value of *g*, $\Vert g-f\Vert <r$, and $g^{-1}(0)$ is connected. The constraint $\Vert g-f\Vert <r$ immediately implies that *f* and *g* are homotopic as maps $A\rightarrow {\mathbb {R}}^n\setminus \{0\}$ and $\phi _f=\phi _g$. As *X* is connected, the group $H_{m}(X,\partial X)$ is generated by the fundamental class of *X* and we already know by Lemma 3 that $\phi _f\frown [X]=\phi _g\frown [X]$ equals the image of the fundamental class of $g^{-1}(0)$ in $H_{m-n}(X)$. But if the manifold $g^{-1}(0)$ is connected, then $H_{m-n}(g^{-1}(0))$ is generated by the fundamental class of $g^{-1}(0)$, so its image in $H_{m-n}(X)$ is generated by $\phi _f\frown [X]$. It follows that $U_{m-n}(f,r)$, being the intersection over all *r*-perturbations, cannot contain anything else than multiples of $\phi _f\frown [X]$, and we obtain the desired inclusion

$$\begin{aligned} \phi _f\frown H_{m-n}(X,\partial X)\supseteq U_{m-n}(f,r). \end{aligned}$$

So, it remains to prove that if $n+1\le \dim X\le 2n-3$, *X* is connected and $A=\partial X$, then there exists a smooth strict *r*-perturbation *g* of *f* transverse to 0 such that $g^{-1}(0)$ connected. To show this, we need to introduce additional concepts from differential topology.

*Framed Submanifolds.* Assume that *X* is a smooth *m*-manifold endowed with a Riemannian metric; the results will be independent on the choice of the metric. Let $S\subseteq X$ be a smooth $(m-n)$-submanifold contained in the interior of *X*; for each $x\in S$, the tangent space $T_x X$ decomposes as a direct sum of the tangent space $T_x S$ and the normal space $N_x S$. A framing on *S* is a trivialization of the normal bundle *NS*, in other words, a smooth mapping *T* such that for each $x\in S$, $T(x)=(T_1(x),\ldots , T_n(x))$ is a basis of the normal space $N_x S$.

If $f: X\rightarrow {\mathbb {R}}^n$ has 0 a regular value, then $f^{-1}(0)$ is naturally a framed $(m-n)$-submanifold, $T_i(x)$ being the unique vector in $N_x f^{-1}(0)$ mapped by *df* to $e_i\in {\mathbb {R}}^n$. We will denote these vectors by $f^*(e_i)$. Assume that *W* is a framed $(m-n+1)$-submanifold of *X* with framing $T_2(x),\ldots , T_{n}(x)$ and that $\partial W$ is the boundary of *W*. The existence of collars implies that some neighborhood of $\partial W$ in *W* is diffeomorphic to $\partial W\times [0,1)$ with coordinates (*w*, *t*). The framing of *W* *induces* a framing of its boundary, given by $(T_1(x),\ldots , T_n(x))$ where $T_1(x)$ is the vector $\partial _t$ in the “inwards” direction and $(T_2(x),\ldots , T_n(x))$ the framing of *W* in $x\in \partial W$.

### Lemma 4

Let *X* be a smooth *m*-manifold, $r>0$, $f: X\rightarrow {\mathbb {R}}^n$ be smooth with 0 a regular value of *f*, $A=\partial X=|f|^{-1}(r)$, and $\dim X\le 2n-3$. Let $S\subseteq X$ be a framed boundary-free $(m-n)$-submanifold of *X* disjoint from *A* and assume that there exists a framed $(m-n+1)$-submanifold $W\subseteq X$ disjoint from *A* so that $\partial W=f^{-1}(0)\sqcup S$ and *W* induces the framing of $f^{-1}(0)\sqcup S$.

Then there exists a smooth *g* so that $\Vert g-f\Vert <r$, 0 is a regular value of *g* and $g^{-1}(0)=S$.

We will see that *g* can be even chosen so that the *S*-framing $(T_1(x),\ldots , T_n(x))$ satisfies $T_1(x)=-g^*(e_1)$ and $T_i(x)=g^*(e_i)$ for $i>1$.

### Proof

*Step 1*:* Reduction to the existence of* *h* *homotopic to* *f*, $h^{-1}(0)=S$.

We will construct a smooth map *h* s.t. $h^{-1}(0)=S$ and *h* / |*h*| will be homotopic to *f* / |*f*| as maps from $A\rightarrow S^{n-1}$. This is sufficient, because then we might easily change *h* in a collar of *A* diffeomorphic to $A\times [0,1]$ that is disjoint from $h^{-1}(0)$ to obtain a smooth extension $e: X\rightarrow {\mathbb {R}}^n$ of $f|_A$ that coincides with *h* outside this neighborhood. According to [13, Lem. 3.1] some positive scalar multiple $\chi (x)\,e(x)=:g(x)$ satisfies $\Vert g-f\Vert <r$. The map $\chi$ can be chosen to be smooth: then 0 is a regular value of *g* and $g^{-1}(0)=h^{-1}(0)=S$. In the rest of the proof, we will show how to construct *h*.

*Step 2*:* Constructing a perturbation* ${\tilde{f}}$ *of* *f*.

Let $(T_2,\ldots ,T_n)$ be the framing on *W*, inducing the framing $(T_1,\ldots , T_n)$ on $f^{-1}(0)\sqcup S = \partial W$. On $f^{-1}(0)$, $T_i$ coincides with $f^*(e_i)$. Let $B_\varepsilon ^n$ be a closed neighborhood of $0\in {\mathbb {R}}^n$ consisting of regular values of *f* and let *L* be the closed straight line segment connecting 0 and $-\varepsilon e_1\in {\mathbb {R}}^n$. Then the *f*-preimage of *L* is an $(m-n+1)$-submanifold of *X* with boundary $f^{-1}(\{0,-\varepsilon \}\times \{0\})$, with a framing $(f^*(e_2),\ldots , f^*(e_n))$, where $e_2,\ldots , e_n$ are the normal vectors to *L* for each $x\in L$. By making $\varepsilon$ possibly smaller, we can assume that $f^{-1}(L)\cap W=f^{-1}(0)$, because in a small enough neighborhood *U* of $f^{-1}(0)$, $f_1$ is positive on $(W\setminus f^{-1}(0))\cap U$ (by definition, $(d f_1)(T_1(x))>0$ for $x\in f^{-1}(0)$) and negative on $(f^{-1}(L)\setminus f^{-1}(0))\cap U$. The vector field $T_1(x)$ for $x\in f^{-1}(0)$ is in the tangent space of both *W* and $f^{-1}(L)$; it has the inwards direction wrt. *W* and outward wrt. $f^{-1}(L)$.

Let $V:=f^{-1}(B_\varepsilon ^n)$. The restriction $f|_V$ is transverse to the closed set

$$\begin{aligned} {\mathbb {R}}_0^- e_1:=(-\infty , 0]\times \{0\}\subseteq {\mathbb {R}}^n \end{aligned}$$

by construction. Using a relative version of transversality theorem, the space of smooth functions that coincide with *f* on *V* and are transverse to ${\mathbb {R}}_0^- e_1$ is dense and open in $\{g\in C^\infty (X,{\mathbb {R}}^n):\,g|_V=f|_V\}$ in Whitney $C^1$-topology (this follows from [1, Thm. 19.1]) so there exists an arbitrary small perturbation ${\tilde{f}}$ of *f* that is smooth, transverse to ${\mathbb {R}}_0^- e_1$ and ${\tilde{f}}|_V=f|_V$. Then ${\tilde{f}}^{-1}({\mathbb {R}}_0^- e_1)$ is a smooth $(m-n+1)$-submanifold of *X* with boundary $f^{-1}(0)={\tilde{f}}^{-1}(0)$.

The assumption $m\le 2n-3$ implies that $\dim W+\dim {\tilde{f}}^{-1}({\mathbb {R}}_0^- e_1)=2(m-n+1)<m$, so both *W* and ${\tilde{f}}^{-1}({\mathbb {R}}_0^- e_1)$ have dimension less than one half of $m=\dim X$. Therefore, we can replace ${\tilde{f}}$ by another arbitrary small perturbation, without changing it on *V*, assume that it is transverse to ${\mathbb {R}}_0^- e_1$ and moreover, ${\tilde{f}}({\mathbb {R}}_0^- e_1)$ intersects *W* only in $f^{-1}(0)$. Assume that ${\tilde{f}}$ is close enough to *f* so that $f|_A$ is homotopic to ${\tilde{f}}|_{A}$ as maps from *A* to ${\mathbb {R}}^n\setminus \{0\}$. Without loss of generality, we may assume that ${\tilde{f}}^{-1}({\mathbb {R}}_0^- e_1)$ intersects *A* transversally (otherwise we replaced ${\tilde{f}}$ by another perturbation that differs from ${\tilde{f}}$ in a neighborhood of *A*) and hence ${\tilde{f}}^{-1}({\mathbb {R}}_0^- e_1)\cap A$ is an $(m-n)$ dimensional submanifold of *A*.

The submanifold ${\tilde{f}}^{-1}({\mathbb {R}}_0^- e_1)$ is endowed with a framing $({\tilde{f}}^*(e_2), \ldots {\tilde{f}}^*(e_n))$ where $e_2,\ldots , e_n$ are vectors of the canonical basis in $T_{(y,0)} {\mathbb {R}}^n$ for $y\le 0$. This framed manifold intersects *W* in $f^{-1}(0)$, the tangent spaces of both manifolds coincide in $f^{-1}(0)$, $T_1$ directs inwards wrt. *W* and outwards wrt. ${\tilde{f}}^{-1}({\mathbb {R}}_0^- e_1)$, and the framing on both submanifolds coincide in $f^{-1}(0)$ (Fig. 3).

*Step 3*:* Gluing* *W* *and* ${\tilde{f}}^{-1}({\mathbb {R}}_0^-\,e_1)$ *to one smooth submanifold.*

Both submanifolds *W* and ${\tilde{f}}^{-1}({\mathbb {R}}_0^- e_1)$ of *X* intersect in their common boundary $f^{-1}(0)$ and both the tangent spaces and framings coincide in $f^{-1}(0)$. We would like to smoothly “glue” them to one framed manifold $W\cup {\tilde{f}}^{-1}({\mathbb {R}}_0^- e_1)$ but unfortunately, such union does not need to yield a *smooth* submanifold in general.

We claim that there exists a smooth framed manifold $W'$ that coincides with $W\cup {\tilde{f}}^{-1}({\mathbb {R}}_0^- e_1)$ everywhere except on a neighborhood of $f^{-1}(0)$ in *X* that can be chosen to be arbitrary small. Choose a continuous tangent vector fields *v* in $W\cup {\tilde{f}}^{-1}({\mathbb {R}}_0^- e_1)$ that is smooth on *W* and smooth on ${\tilde{f}}^{-1}({\mathbb {R}}_0^- e_1)$, such that $v|_{f^{-1}(0)}$ is nonzero and points inwards wrt. *W* and outwards wrt. ${\tilde{f}}^{-1}({\mathbb {R}}_0^- e_1)$ (it may coincide with $f^*(e_1)$). The flow of this vector fields induces collar neighborhoods $C_1$ resp. $C_2$ of $f^{-1}(0)$ in *W* resp. ${\tilde{f}}^{-1}({\mathbb {R}}_0^- e_1)$ contained in *X* diffeomorphic to $f^{-1}(0)\times [0,\varepsilon )$, resp. $f^{-1}(0)\times (-\varepsilon ,0]$ for some $\varepsilon >0$. Let as denote the embeddings $f^{-1}(0)\times [0,\varepsilon )\rightarrow X$ and $f^{-1}(0)\times (-\varepsilon , 0]\rightarrow X$ by $w_1$ and $w_2$, respectively. Let $w: f^{-1}(0)\times (-\varepsilon ,\varepsilon )\rightarrow X$ be defined by $w_1(x,t)$ for $t\ge 0$ and $w_2(x,t)$ for $t<0$: this map is a $C^1$-embedding (the differentials $dw_1$ and $dw_2$ coincide on $f^{-1}(0)$) and is $C^\infty$ whenever $t\ne 0$.

Let $\psi _\alpha : U_\alpha \rightarrow {\mathbb {R}}^m$ be a collection of *X*-charts and let $\{V_\beta \}_\beta$ be an open covering of $f^{-1}(0)\times [-\varepsilon /2, \varepsilon /2]$ such that for each $V_\beta$, $w(\overline{V_{\beta }})$ is contained in some $U_{\alpha (\beta )}$. Further, let $V_\beta '\subseteq V_\beta$ be so that $\overline{V_\beta '}\subseteq V_\beta$ and $\{V_\beta '\}_\beta$ is still an open covering of $f^{-1}(0)\times [-\varepsilon /2, \varepsilon /2]$. Let $\beta _1,\ldots , \beta _k$ be such that $\cup _j V_{\beta _j}'$ is an open neighborhood of $f^{-1}(0)\times \{0\}$. The space $C^\infty (\overline{V_{\beta _1}}, {\mathbb {R}}^m)$ is dense in $C^1(\overline{V_{\beta _1}}, {\mathbb {R}}^m)$ with the Whitney $C^1$-topology [20, Thm. 2.4] so we may replace $\psi _{\alpha (\beta _1)}\circ w|_{\overline{V_{\beta _1}}}: \overline{V_{\beta _1}}\rightarrow {\mathbb {R}}^m$ by an arbitrary close map (in the Whitney topology) $\overline{V_{\beta _1}}\rightarrow {\mathbb {R}}^m$ that is smooth on $V_{\beta _1}'$ and unchanged in a neighborhood of $\partial V_{\beta _1}$. This defines a map $w_1': f^{-1}(0)\times [-\varepsilon /2,\varepsilon /2]\rightarrow X$ that is smooth on $V_{\beta _1}'$ and coincides with *w* on $f^{-1}(0)\times \{\pm \varepsilon /2\}$. On $w_1'(\overline{V_{\beta _1}})$, we can also use the $\psi _{\alpha (\beta _1)}$-chart to define a framing that is smooth on $w_1(V_{\beta _1}')$ and coincides with the original framing on a neighborhood of $w_1'(\partial V_{\beta _1})$. In the same way, we smoothen the function on $V_{\beta _2}',\ldots , V_{\beta _k}'$ and obtain a smooth map $w': f^{-1}(0)\times (-\varepsilon , \varepsilon )\rightarrow X$ arbitrary close to *w* that coincides with *w* on a neighborhood of $f^{-1}(0)\times \{\pm \varepsilon \}$. If we chose $w'$ close enough to *w*, it is an embedding by [20, Thm. 1.4]. The manifold $W'$ can now be defined as

$$\begin{aligned} W':=\mathrm {Im}(w')\cup \big ((W\cup {\tilde{f}}^{-1}({\mathbb {R}}_0^- e_1))\setminus \mathrm {Im}(w')\big ), \end{aligned}$$

which is a smooth embedded framed submanifold that coincides with $W\cup {\tilde{f}}^{-1}({\mathbb {R}}_0^- e_1)$ except on a neighborhood of $f^{-1}(0)$ that can be chosen to be arbitrary small. The framing coincides with the framing on $W\cup {\tilde{f}}^{-1}({\mathbb {R}}_0^- e_1)$ outside $\mathrm {Im}(w')$. By construction, the boundary of $W'$ consists of *S* and a submanifold $W'\cap A$ of *A*.

*Step 4*:* Choice of the metric.*

Let us choose a vector field *v* on $A=\partial X$ in the inwards-direction so that for $x\in W'\cap A$, $v(x)\in T_x W'$. This can be extended to a nowhere zero vector field in a neighborhood of *A* and used to define a collar neighborhood of *A* diffeomorphic to $A\times [0,\varepsilon )$ for some $\varepsilon >0$, the diffeomorphism induced by the flow of *v*. We endow *X* with a new smooth Riemannian metric that is a product metric on this neighborhood. Due to this choice of the metric, the geodesics in *A* coincide with the geodesics in *X*. In what follow, we will assume that such metric has been defined and we identify the given framing of $W'$ and *S* with normal vectors wrt. this metric. In particular, $W'$ intersects *A* orthogonally and the $W'$-framing vectors in $W'\cap A$ are all in the tangent space of *A*.

*Step 5*:* Construction of* *h*.

The first framing vector $T_1$ of the *S*-framing can be extended to a smooth tangent (wrt. *W*) vector field in a neighborhood of *S* in *W* and further to a neighborhood of *S* in *X*. The flow of $-T_1$ then generates an external collar *C* of $W'$ diffeomorphic to $S\times [0,\varepsilon ]$ for some $\varepsilon >0$ such that $W'':=C\cup W'$ is a smooth submanifold of *X*. Using charts and partition of unity, we may easily extend the $W'$-framing to a framing on $W''$. Without loss of generality, we can assume that the external collar *C* of *W* is disjoint from *A*. The flow of $-T_1$ induces a neighborhood $\nu (S)$ of *S* in $W''$ diffeomorphic to $S\times [-\varepsilon ,\varepsilon ]$ with $S\times (-\varepsilon ,0]$ corresponding to a neighborhood of *S* in $W'$ and $S\times [0,\varepsilon )$ to the open external collar of *S* contained in $W''$. The projection on $[-\varepsilon , \varepsilon ]$ defines a smooth scalar valued map $h_1: \nu (S)\rightarrow {\mathbb {R}}$ s.t. $h_1^{-1}(0)=S$, $h_1\ge 0$ on *C* and $h_1\le 0$ on $\nu (S)\cap W'$: $(h_1)_*$ maps $T_1$ (which directs inwards to *W*) to $(-\partial _x)\in T_0{\mathbb {R}}$.

By construction, ${\tilde{f}}_1$ is negative in $W''\cap A$. Let *U* be a closed neighborhood of *A* in *X* such that ${\tilde{f}}_1$ is still negative on $U\cap W''$ and extend $h_1$ to a smooth map $W''\rightarrow {\mathbb {R}}$ such that $(h_1)|_{U\cap W''}=({\tilde{f}}_1)|_{U\cap W''}$ and $h_1<0$ on $W'\setminus S$.

The geodesic flow of the $W''$-framing of $W''$ induces a diffeomorphism $\varphi$ of $W''\times B^{n-1}$ and some set $\nu (W'')\subseteq X$, where $B^{n-1}$ is the closed ball in ${\mathbb {R}}^{n-1}$ of small enough diameter (due to our choice of the metric, framing vectors in $W'\cap A$ induce geodesics in *A*). This set $\nu (W'')$ is not open in *X*, but it contains an open neighborhood of $W'$. The projection on $B^{n-1}$ defines a smooth function $h': \nu (W'')\rightarrow {\mathbb {R}}^{n-1}$ transverse to 0 such that $h'^{-1}(0)=W''$ and $h'$ induces the given framing on $W''$. Let us extend $h_1$ to a smooth scalar valued map $\nu (W'')\rightarrow {\mathbb {R}}$ arbitrarily and finally define $h: \nu (W'')\rightarrow {\mathbb {R}}^n$ by $h=(h_1, h')$. It is easy to see that $h^{-1}(0)=S$ and 0 is a regular value of *h*. Summarizing the construction, we have a (closed) neighborhood $\nu (W'')$ of $W'$ in *X* and a smooth map $h: \nu (W'')\rightarrow {\mathbb {R}}^n$ such that

- $h^{-1}(0)=S$, the original framing of *S* equals $(-h^*(e_1), h^*(e_2),\ldots h^*(e_n))$,
- $(h_2,\ldots , h_n)^{-1}(0)=W''$ and $(h_2,\ldots , h_n)$ induces the original framing on $W'\subseteq W''$,
- $$W''\cap h^{-1}({\mathbb {R}}_0^- e_1)=W'$$,
- $$h|_{U\cap W''}={\tilde{f}}|_{U\cap W''}$$.

By construction, *h* restricted to $\partial (\nu (W''))$ has values in ${\mathbb {R}}^n\setminus \{{\mathbb {R}}_0^-\,e_1\}$: this is because $h(x)\in {\mathbb {R}}_0^- e_1$ implies $x\in W'$ and $W'$ is in the interior of $\nu (W')$. The topological space ${\mathbb {R}}^n\setminus ({\mathbb {R}}_0^- e_1)$ is homotopically trivial as it deformation retracts to a point, so $h|_{\partial \nu (W'')}$ can be extended to a continuous map $\overline{X\setminus \nu (W'')}\rightarrow {\mathbb {R}}^n\setminus \{{\mathbb {R}}_0^-\,e_1\}$ (for this, we need the homotopy extension property of $\overline{X\setminus \nu (W'')}$ and its closed subset $\partial \nu (W'')$) which in turn defines an extension $h: X\rightarrow {\mathbb {R}}^n$ of the map $h|_{\nu (W'')}$ that we have already defined. Possibly perturbating *h* slightly outside of some neighborhood of $W''$, we may assume that it is smooth [22, Thm. 2.5]. By construction, 0 is a regular value of *h*.

*Step 6*:* The restriction* $h|_A$ *is homotopic to* $f|_A$.

We will show that $H:=h/|h|$ and $F:=f/|f|$ are homotopic as maps from $A\rightarrow S^{n-1}$. Let ${\tilde{F}}$ be the restriction of $({\tilde{f}}/|{\tilde{f}}|)$ to the open neighborhood *U* of *A*. We assumed that $f|_{A}$ is homotopic to ${\tilde{f}}|_{A}$ as maps $A\rightarrow {\mathbb {R}}^n\setminus \{0\}$, so the sphere-valued maps $F|_{A}$ is homotopic to ${\tilde{F}}|_{A}$ and it remains to show that ${\tilde{F}}|_{A}$ is homotopic to $H|_{A}$.

By construction, $({\tilde{f}}|_{U})^{-1}({\mathbb {R}}_0^- e_1)=({h|_U})^{-1}({\mathbb {R}}_0^- e_1)=U\cap W''$, both maps are nowhere zero on *U*, they coincide on $U\cap W''$ and both maps induce the same framing on $U\cap W''$. It follows that ${\tilde{F}}^{-1}(-e_1)=(H|_U)^{-1}(-e_1)=U\cap W''$ and both ${\tilde{F}}$ and *H* induce the same framing on $U\cap W''$ (it coincides with the original framing of $U\cap W''$ up to scalar multiples of framing vectors): this framing restricts on $W''\cap A$ to a framing of the normal space to $W''\cap A$ in *A*. Therefore, for $x\in {\tilde{F}}^{-1}(-e_1)\cap A$, ${\tilde{F}}_*(x)=H_*(x)$ and consequently $({\tilde{F}}|_{A})_*(x)=(H|_{A})_*(x)$. It follows that ${\tilde{F}}|_{A}$ and $H|_{A}$ induce the same framing of the normal bundle $N(({\tilde{F}}|_{A})^{-1}(-e_1)\cap A)$ in *A* and by [25, Lem. 4, p. 48], ${\tilde{F}}|_{A}\sim H|_{A}$ are homotopic. $\square$

*Connecting Disconnected Components.* In this section, we show that if $S_1$ is a framed submanifold of *X* with dimension at least 1 and codimension at least 3, then there exists a framed submanifold $W\subseteq X$ such that $\partial W=S_1\sqcup S_2$ where $S_2$ is connected. This will finish the proof of Theorem 3, because it follows that for the framed submanifold $S_1:=f^{-1}(0)$, we can construct a strict *r*-perturbation *g* of *f* s.t. $g^{-1}(0)=S_2$ is connected by Lemma 4. The constraint $n+1\le m\le 2n-3$ that we assume in Theorem 3 implies that the dimension of $f^{-1}(0)$ is at least 1 and that $n\ge 4$, so all the dimensional assumptions of Lemmas 5 and  4 are satisfied.

### Lemma 5

Let *X* be a smooth connected manifold, $S_1$ a framed closed submanifold of *X* 27 and assume that $1 \le \dim S_1\le \dim X-3$. Then there exists a framed submanifold *W* in the interior of *X* such that $\partial W=S_1\sqcup S_2$, *W* induces the framing on $S_1\sqcup S_2$ and $S_2$ is connected.

The main idea of the proof is to construct a manifold $W_1\simeq S_1\times [0,1]$, cut out two holes in $S_1\times \{1\}$ around *x* and *y* that are in different components of $S_1\times \{1\}$ and connect them with a tubular $(\dim S_1+1)$-dimensional neighborhood of a curve connecting *x* and *y*. While there is a well-known construction called “boundary connected sum” for abstract differential manifolds [22], we could not find any reference that this can be done all inside the ambient space *X*, so here we present the sketch of our construction.

### Proof

Let $m:=\dim X$ and $n:=\dim X-\dim S_1$. By the product neighborhood theorem, the framing of $S_1$ determines a diffeomorphism *d* from $S_1\times B_2^n$ to a (closed) neighborhood *U* of $S_1$ in *X*, where $B_2^n\subseteq {\mathbb {R}}^n$ is the closed ball of diameter 2. Let as choose a smooth metric on $S_1$, extend it to a product metric on *U* via the diffeomorphism *d* and smoothly extend it to a metric on the whole of *X*. Let $v_1,\ldots , v_n$ be the vector fields on *U* defined as the $d_*$-image of the euclidean coordinate vector fields $e_1,\ldots , e_n\in TB_2^n$ identified with vectors of $T(S_1\times B_2^n)\simeq TS_1\times TB_2^n$ orthogonal to $TS_1\times \{0\}\subseteq T_{(s,b)} (S_1\times B_2^n)$. The image $d(S_1 \times [0,1]\times \{0\})=:W_1$ is a smooth submanifold of *X* of dimension $m-n+1$ contained in *U*, with boundary $\partial W_1=S_1\sqcup S_1'$ where $S_1'=d(S_1\times \{1\}\times \{0\})$. The vectors $v_2,\ldots , v_n$ form a framing of $W_1$ and the vector field $(v_1)|_{W_1}$ is tangent to $W_1$ such that on the boundary, $v_1|_{S_1}$ goes in the “inwards” and $v_1|_{S_1'}$ in the “outwards” direction wrt. $W_1$. Further, $S_1'$ is a diffeomorphic copy of $S_1$, so it has the same number of connected components. Let $x,y\in S_1'$ be two points in different components of $S_1'$ (Fig. 4).

Let $\varphi : [0,1]\rightarrow X$ be a smooth embedded curve such that $\varphi (0)=x$, $\varphi (1)=y$, $\varphi (t)\notin W_1$ for $t\ne 0,1$ and there exists an $\delta >0$ such that ${\dot{\varphi }}(t)=v_1(\varphi (t))$ for $t\in [0,\delta ]$ and ${\dot{\varphi }}(t)=-v_1(\varphi (t))$ for $t\in [1-\delta ,1]$. Such curve exists, because *X* is connected and the codimension of $W_1$ in *X* is at least two, so $X\setminus W_1$ is still connected. Without loss of generality, we may assume that $\delta$ is small enough so that $\varphi ([0,\delta ]\cup [1-\delta , 1])$ is in the image of $d: S_1\times B_2^n\rightarrow U$.

The curve $\varphi [0,1]$ is contractible, so any fibre bundle over it is trivial and admits a global section; in particular, there exists a global section *T* of the principal bundle of all framings of its $m-1$-dimensional normal bundle. That means, for $x=\varphi (t)$, $T(x):=(u_1,\ldots , u_{m-1})$ is a framing of $N_x \varphi [0,1]$ and *T* is smooth. Any other framing of $\varphi [0,1]$ is determined by a smooth map $[0,1]\rightarrow Gl(m-1)$ which linearly transforms the framing vectors in each $\varphi (t)$. Let $w_1,\ldots ,w_{m-n}$ be a basis of $T_x S_1'$, resp. $T_y S_1'$, oriented so that $(w_1(x),\ldots , w_{m-n}(x), v_2(x),\ldots , v_n(x))$ has the same orientation of $N_x \varphi [0,1]$ as *T*(*x*) and $(w_1(y),\ldots , w_{m-n}(y), v_2(y),\ldots , v_n(y))$ has the same orientation of $N_y \varphi [0,1]$ as *T*(*y*). Let as naturally extend the vector fields $w_1,\ldots , w_{m-n}$ to $\varphi ([0,\delta ]\cup [1-\delta , 1])$ by means of parallel transport along $\varphi$. The connectedness of $Gl^+(m-1)$ implies that there exists a framing $T'$ of $N(\varphi [0,1])$ such that $T'(\varphi (t))$ coincides with $(w_1,\ldots , w_{m-n}, v_2,\ldots , v_n)$ for $t\in [0,\delta ]\cup [1-\delta , 1]$. By a slight abuse of notation, we again denote the first $m-n$ framing vectors of $T'$ by $w_1,\ldots , w_{m-n}$: these are normal vector fields on $\varphi [0,1]$ extending the already defined $\{w_i\}_i$ in $\varphi ([0,\delta ]\cup [1-\delta , 1])$.

For any $a\in \varphi [0,1]$ and $u\in {\mathbb {R}}^{m-n}$, let *F*(*a*, *u*) be equal to $\gamma (1)$ where $\gamma$ is a geodesic, $\gamma (0)=a$ and ${{\dot{\gamma }}}(0)=u_1 w_1+u_2 w_2+\ldots +u_{m-n} w_{m-n}$ whenever the geodesic is defined on [0, 1]. If $\varepsilon >0$ is small enough, then $F: \varphi [0,1]\times B_\varepsilon ^{m-n}\rightarrow X$ is a smooth embedding and its image is an embedded $(m-n+1)$-dimensional submanifold of *X* (with corners in $\{x,y\}\times \partial B_\varepsilon ^{m-n}$). Using the properties of our metric, the $S_1'$-geodesics in *x*, resp. *y* coincide with the geodesics in *X*, so *F* maps $\{x,y\}\times B_\varepsilon ^{m-n}$ to a closed neighborhood $D_x\sqcup D_y$ of $\{x, y\}$ in $S_1'$, where $D_x$ resp. $D_y$ is a geodesic $\varepsilon$-ball in $S_1'$.

If $t<\delta$ or $t>1-\delta$, then $F(\varphi (t),B_{\varepsilon }^{m-n})$ is contained in *U* and disjoint from $W_1$ due to the choice of the product metric. If $t\in [\delta ,1-\delta ]$, then there exists some $\varepsilon (t)<\varepsilon$ and a neighborhood *U*(*t*) of *t* such that $F(\varphi (U(t)),B_{\varepsilon (t)}^{m-n})$ is disjoint from $W_1$. By compactness of [0, 1], we can make $\varepsilon$ smaller and assume that $F(\varphi (0,1)\times B_\varepsilon ^{m-n})$ is disjoint from $W_1$. Let $T:=F(\varphi [0,1]\times B_\varepsilon ^{m-n})$. This is a smooth contractible $(m-n+1)$-manifold (with corners), so its normal bundle admits a global framing. By an argument completely analogous to the one above, we may extend the framing $v_2,\ldots , v_n$ defined on $F(\varphi ([0,\delta ]\cup [1-\delta , 1])\times B_\varepsilon ^{m-n})$ to a smooth framing of *T*.

By construction, $T\cap W_1$ consists of two $(m-n)$-discs $D_x=F(\{x\}\times B_\varepsilon ^{m-n})$ and $D_y$ in $S_1'$. At this point, $W_1\cup T$ is a framed manifold, but we still need to “smooth the corners” $\partial D_x$ and $\partial D_y$.

Let $\psi : [0,1]\rightarrow [\frac{1}{2}\varepsilon , \varepsilon ]$ be a smooth function such that $\psi (0)=\psi (1)=\varepsilon$, $\psi '(0)=-\infty$, $\psi '(1)=\infty$ and further, for some $\beta >0$, the inverse function $(\psi |_{[0,\beta ]})^{-1}: [\psi (\beta ),\varepsilon ]\rightarrow [0,\beta ]$ can be extended to a *smooth* function $[\psi (\beta ), \infty )\rightarrow [0,\beta ]$ by sending each $x>\varepsilon$ to 0, and similarly $(\psi |_{[1-\beta ,1]})^{-1}: [\psi (1-\beta ),\varepsilon ]\rightarrow [1-\beta ,1]$ can be extended to a *smooth* function defined on $[\psi (1-\beta ),\infty )$ by mapping each $x>\varepsilon$ to 1.^28^ Finally, define $T'\subseteq T$ by

$$\begin{aligned} T':=\{F(\varphi (t),u):\,|u|\le \psi (t)\}. \end{aligned}$$

We claim that $W:=W_1\cup T'$ is a smooth manifold with boundary. By construction, $W_1$ and $T'\setminus W_1$ are smooth manifolds with $W_1\cap T'=D_x\cup D_y$, so we just need to analyze their intersection (Fig. 5).

Let *u* be in the interior of $D_x$, resp. $D_y$. Let *V* be an open neighborhood of *v* with positive distance from $\partial D_x$ (resp. $\partial D_y$) and define an $S_1'$-chart $\phi _u: V\rightarrow {\mathbb {R}}^{m-n}$ that maps a neighborhood $V\subseteq D_x$ (resp. $D_y$) of *u* to ${\mathbb {R}}^{m-n}$. Let $N\subseteq X$ be a neighborhood of *v* in *X* that is disjoint from the topological boundary of *W* in *X* and $N\cap S_1'\subseteq V$. Then the diffeomorphism $(\phi _u, \mathrm {id})\circ d^{-1}$ takes *N* to an open subset of ${\mathbb {R}}^m$ such that $N\cap W$ is the preimage of ${\mathbb {R}}^{m-n+1}\times \{0\}$ (the projection to the $(m-n+1)$’th component of the image of *N* is a neighborhood of $1\in {\mathbb {R}}$).

It remains to show that any $v\in \partial D_x$ resp. $\partial D_y$ is in the boundary of $W=T'\cup W_1$, that is, some neighborhood of *v* in *W* is mapped by an *X*-chart to ${\mathbb {R}}^{m-n}\times (-\infty ,1]\times \{0\}$. Let $\phi _v$ be an $S_1'$-chart mapping a neighborhood *V* of *x* in $S_1'$ to ${\mathbb {R}}^{m-n}$ such that $u_v(v)=0$ and let $N:=d^{-1}(V\times (0,1+\delta ))\subseteq X$ be a neighborhood of *v* in *X*. Let $(\phi _v, \hbox {id})\circ d^{-1}: N\rightarrow {\mathbb {R}}^m$ be an *X*-chart: it maps

- $$S_1'\cap N$$ to $${\mathbb {R}}^{m-n}\times \{1\}\times \{0\}$$,
- $$W_1\cap N$$ to $${\mathbb {R}}^{m-n}\times (0, 1]\times \{0\}$$,
- $$T'\cap N$$ to $$\{(z_1,\ldots , z_{m-n}, y, 0,\ldots , 0):\,\,1\le y\le \omega (z)$$}, and
- $$W\cap N$$ to $$\{(z_1,\ldots , z_{m-n}, y, 0,\ldots , 0):\,\,0\le y\le \omega (z)\}$$

where $\omega (x)=1$ whenever $x\notin D_x$ and $\omega (z)=\sup \,\{t+1:\,d(z,t,0)\in T'\}$.

If *e*(*u*) is the geodesic distance of *u* from *x*, then $\omega (z)=\sup \,\{t+1:\,e(z)\le \psi (t)\}$. The function $\psi$ maps a neighborhood of 0 to some $(\varepsilon -\alpha , \varepsilon ]$ and the inverse function $\psi ^{-1}$ to this restriction has derivative 0 in $\varepsilon$. If we extend $\psi ^{-1}(a)$ to be 0 for $a>\varepsilon$, we get a smooth function from a neighborhood of $\varepsilon$ in ${\mathbb {R}}$ to nonnegative numbers with $\psi ^{-1}(\varepsilon )=(\psi ^{-1})'(\varepsilon )=0$. We can rewrite $\omega (z)$ to $\psi ^{-1}(e(z))+1$ to see that it is a smooth function defined on a neighborhood of *v* in $S_1'$ with zero gradient in *v* (note that $e(v)=\varepsilon$). It follows that $W\cap N$ is diffeomorphic to some neighborhood of $(0,\ldots , 0,1,0,\ldots , 0)$ in $\{(z_1,\ldots , z_{m-n}, y, 0,\ldots , 0):\,\,y\le \omega (z)\}$ which is diffeomorphic to ${\mathbb {R}}^{m-n}\times (-\infty , 0]\times \{0\}$.

Hence *W* is a smooth framed submanifold of *X* of dimension $m-n+1$, the framing of the normal bundle being a restriction of the framing of $W_1$ and *T*. If $m-n\ge 2$, then $\dim \partial D_x\simeq S^{m-n-1}$ is a sphere of dimension at least one and hence is connected: by construction, $\partial D_x$ and $\partial D_y$ are in the same component of the boundary $\partial W$. If $m-n=1$, then $S_1'$ is a finite disjoint union of circles and yields $\partial W$ to be a connected sum of two circles containing *x* and *y* united with $S_1$ and other components of $S_1'$. In both cases, the number of connected components of $\partial W\setminus S_1$ is smaller then the number of connected components of $S_1'$. The compactness of *X* and $S_1$ implies that $S_1$ has only a finite number of components. In the same way as above, we may continue attaching tubular neighborhoods of curves connecting different components of $S_1'$ to obtain a framed manifold *W* such that $\partial W\setminus S_1=:S_2$ is already connected. $\square$

## Appendix 3: Proof of Theorem 5

We need the following observation, cf. [5, p. 9].

### Lemma 6

Let (*X*, *A*) be an *m*-dimensional pair of simplicial complexes such that *A* contains the $(i-1)$-skeleton of *X*. Then there is an $(m-i)$-subcomplex *Y* of a subdivision $X'$ of *X* such that *Y* is disjoint with *A* and $X'\subseteq A*Y$.

Moreover, for each $(m-i)$-simplex $\tau$ in *Y* there is an *i*-simplex $\sigma$ of $X\setminus A$ such that the following holds: $\sigma '*\tau$ is a simplex of $X'$ if and only if $\sigma '$ is a face of $\sigma$.

The subcomplex *Y* from the previous lemma is called the *dual complex to* *A* *in* *X*. Also the simplex $\sigma$ is called the *dual simplex to* $\tau$.

### Proof

We proceed by induction on the dimension of *X*. When $m=i-1$, the statement holds for the empty subcomplex *Y* of *X*.

When $m\ge i$ we can use the induction hypothesis on $X^{(m-1)}$ to obtain the dual complex $Y^{(m-i-1)}\subseteq X'^{(m-1)}$ where $X'^{(m-1)}$ is a subdivision of $X^{(m-1)}$. For each *m*-simplex $\sigma$ of $X\setminus A$ we have that $\sigma \cong \partial \sigma * b_\sigma$ where $b_\sigma$ is the barycenter of $\sigma$. Since $\partial \sigma$ can be considered as a subcomplex of $X'^{(m-i-1)}$, we have that $X^{(m-1)}\cup \sigma$ is a subspace of $X'^{(m-1)} * b_\sigma$. Similarly whole *X* is a subspace of $X'^{(m-i)}* \{b_\sigma :\sigma \hbox { is an m-simplex of }X\}$. Therefore the desired *Y* can be set to $Y^{(m-i-1)} * \{b_\sigma :\sigma \hbox { is an m-simplex of }X\}$.

To prove the second statement, we need to distinguish two cases. First, when $m=i$, then the dual simplex to each $[b_\sigma ]$ is $\sigma$. Second, when $m>i$, each $(m-i)$-simplex $\tau$ has the form $b *\tau '$ for some $(m-i-1)$-simplex $\tau '$ of $Y^{(m-i-1)}$. The dual simplex $\sigma$ to $\tau$ is equal to the dual simplex of $\tau '$ from the induction since $\sigma ' *\tau \in X'$ if and only if $\sigma ' * \tau '\in X'^{(m-1)}$. $\square$

Observation 1 directly follows from the following lemma:

### Lemma 7

Let $f:K\rightarrow {\mathbb {R}}^n$ be a map such that $(X,A):=|f|^{-1}([0,r], \{r\})$ is pair of simplicial complexes. If the map $f|_A$ can be extended to a map $f^{(i-1)}:X^{(i-1)}\cup A\rightarrow S^{n-1}$, then there is an *r*-perturbation *g* of *f* such that $g^{-1}(0)$ is an $(m-i)$-dimensional subcomplex of some subdivision of *X*.

### Proof

To prove the lemma we need to find an extension $g:X\rightarrow {\mathbb {R}}^n$ of a given map $f^{(i-1)}:X^{(i-1)}\cup A\rightarrow S^{n-1}$ such that $g^{-1}(0)$ is a simplicial complex of dimension at most $m-i$. Let *Y* be the dual complex to $X^{(i-1)}\cup A$ in *X*. We define the extension $g:X\rightarrow {\mathbb {R}}^n$ by $g:=f*{\varvec{0}}_Y$, that is, for each point (*x*, *t*, *y*) of each simplex $\sigma *\tau$ in $X\subseteq (X^{(i-1)}\cup A)* Y$ we define $g(x,t,y):=tf(x)+(1-t)0$. Clearly, $g^{-1}(0)=Y$. $\square$

### Proof of Theorem 5

We are given an *m*-dimensional simplicial pair (*X*, *A*), natural numbers *i* and *n* such that $n<i\le (m+n)/2-1$ and a map $h:X^{(i-1)}\cup A\rightarrow S^{n-1}$. To prove the theorem, we will find an extension $g:X\rightarrow {\mathbb {R}}^n$ of *h* such that $g^{-1}(0)$ is a cell complex obtained from an $(m-i-1)$-dimensional simplicial complex by attaching cells of dimension $m-n$.

We will use the dual complex *Y* to $$X^{(i-1)}\cup A$$ in *X* as in Lemma 6. Part of the map *g* is easy to define, namely, for every $$(x,t,y) \in (X^{(i-1)}\cup A)*Y^{(m-i-1)}$$ we set $$g(x,t,y):=(1-t) h(x)$$. (Note that for $$t=1$$, we have $$(x,t,y)\in Y^{(m-i-1)}$$.) For the rest, we need to define *g* on each $$ {\Delta }:=(\partial \sigma ) * \tau $$ where $$\tau $$ is an arbitrary $$(m-i)$$-simplex of *Y* and $$\sigma $$ is its dual *i*-simplex in $$X\setminus A$$. We have that $${\Delta }=\partial \sigma *(b_\tau * \partial \tau )=(\partial \sigma *b_\tau )*\partial \tau $$ where $$b_\tau $$ is the barycenter of $$\tau $$. Therefore we can write each point *p* of the join $$( \partial \sigma *b_\tau )*\partial \tau $$ as $$p=(x,s,t,y)$$ where $$x\in \partial \sigma $$, $$y\in \partial \tau $$ and $$s,t\in [0,1]$$. (We have $$p\in \partial \sigma $$ for $$s=0$$ and $$p\in \tau $$ for $$t=1.)$$

1. In the case where $h|_{\partial \sigma }$ is homotopically nontrivial, we define
  $$\begin{aligned} g(x,s,t,y):=(1-t)\big ((1-s)h(x)-{ts} \iota (y)\big ), \end{aligned}$$
  where $\iota :\partial \tau \cong S^{m-i-1}\rightarrow S^{n-1}$ is an embedding of $\partial \tau$ to the equatorial $(m-i-1)$-subsphere. Here we need that $m-i\le n$.
2. Otherwise, we choose an arbitrary extension $h':(\partial \sigma )*b_\tau \rightarrow S^{n-1}\subseteq {\mathbb {R}}^n$ of $h|_{\partial \sigma }$ and define
  $$\begin{aligned} g(x,s,t,y):=(1-t) h'(x,s). \end{aligned}$$
  We can see that ${(g|_{{\Delta }}})^{-1}(0)$ is equal to $\partial \tau$ in this case.

To finish the proof, it suffices to show that in the case 1 above, ${(g|_{{\Delta }}})^{-1}(0)\cong {{\mathrm{Cone}}}(\eta )$ for some $\eta :S^{m-n-1}\rightarrow \partial \tau \cong S^{m-i-1}$. Roughly speaking, we need to solve the equation $h|_{\partial \sigma }(x)=\iota (y)$, that is, to identify the $(h|_{\partial \sigma })$-preimage of equatorial $(m-i-1)$-subsphere of $S^{n-1}$. Informally, our strategy is to employ the fact that the elements $[h|_{\partial \sigma }]$ of the stable homotopy group $\pi _{i-1}(S^{n-1})$ are iterated suspensions and thus, without loss of generality, the $(h|_ {\partial \sigma })$-preimage of the equatorial $(m-i-1)$-subsphere is the equatorial subsphere of the same codimension (and the map $\eta$ above is the restriction of *h* onto this subsphere).

- Formally, by the Freudenthal suspension theorem we know that $[h|_{\partial \sigma }]$ equals a *j*-fold suspension ${\varSigma }^j [\eta ]$ for some $\eta :S^{i-1-j}\rightarrow S^{n-1-j}$ assuming the condition $i-1-j\le 2(n-1-j)-1$. Given the requirement $n-1-j=m-i-1$, the condition is equivalent to $i\le (m+n)/2-1$—the assumption of the theorem.
- Without loss of generality, we can assume that $h|_{\partial \sigma }={\varSigma }^j\eta$. Indeed, in general, there is a homotopy $H:h|_{ \partial \sigma }\sim {\varSigma }^j \eta$. We can parameterize a regular neighborhood *N* of $\partial \sigma$ in $(\partial \sigma *b_\tau )$ by $N\cong \partial \sigma \times [0,1)$. Since ${\Delta }\setminus (N* \partial \tau ) \cong {\Delta }$, the map *g* can be defined on the domain ${\Delta }\setminus (N*\partial \tau )$ via the same formula as above. For each point (*x*, *s*, *t*, *y*) of $N*\partial \tau$ we define $g(x,s,t,y):=\alpha H(s,t)$.
- Now it is easy to see that
  $$\begin{aligned} {(g|_{{\Delta }}}){-1}(0)=\Big \{ \Big (x, s, {1-s\over s}, \eta (x)\Big )\in {\Delta }: x\in S^{m-n-1}\hbox { and }s\in [0.5,1]\Big \}, \end{aligned}$$
  where $S^{m-n-1}$ denotes the equatorial subsphere of $\partial \sigma \cong S^{i-1}$. Because of the identifications $(x,s,1,y)\sim (x',s',1,y)$ and $(x,1,0,y)\sim (x',1,0,y')$ in the join ${\Delta }=(\partial \sigma *b_\tau ) * \partial \tau$, we get that the zero set is homeomorphic to $\{(x,s)\in S^{m-n-1}\times [0.5,1]\}/_\sim$ where the equivalence $\sim$ is defined by
  $$\begin{aligned} (x,1)\sim (x',1)\hbox { for each x,x' and }(x,0.5)\sim (x',0.5)\hbox { when }\eta (x)=\eta (x'). \end{aligned}$$
  But this space is homeomorphic to ${{\mathrm{Cone}}}(\eta )$ by definition.

$$\square $$

## Appendix 4: Cap Product in Čech (Co)homology

Let (*X*, *A*) be a pair of topological spaces and denote $H_*(X,A)$ ($H^*(X,A)$) the Čech (co)homology of (*X*, *A*); we assume a fixed coefficient group *G* for the homology and the constant sheaf *G* for the cohomology. If ${\mathcal {U}}$ is a covering of *X* and ${\mathcal {V}}$ a refinement, then ${\mathcal {U}}_A:=\{U\cap A:\,\,U\in {\mathcal {U}}\}$ is a covering of *A* and ${\mathcal {V}}_A$ is its refinement. We associate to ${\mathcal {U}}$ the nerv $N({\mathcal {U}})$ of the covering and further define the nerv $N({\mathcal {U}}_A)$ to be a simplicial complex whose *q*-simplices are all sets $\{U_0,\ldots ,U_q\}\subseteq {\mathcal {U}}$ containing $q+1$ elements such that $(\cap _{j=0}^q U_j)\cap A\ne \emptyset$: this is a subcomplex of $N({\mathcal {U}})$. A map $p: {\mathcal {V}}\rightarrow {\mathcal {U}}$ that maps each set in ${\mathcal {V}}$ to a superset in ${\mathcal {U}}$, induces a map $p_A: {\mathcal {V}}_A\rightarrow {\mathcal {U}}_A$ and maps on the nerves $N({\mathcal {V}})\rightarrow N({\mathcal {U}})$, $N({\mathcal {V}}_A)\rightarrow N({\mathcal {U}}_A)$, which are simplicial maps between simplicial complexes. Any other choice $p': {\mathcal {V}}\rightarrow {\mathcal {U}}$ yields a homotopic map $N({\mathcal {V}})\rightarrow N({\mathcal {U}})$, so a subcovering induces well-defined maps $p_*$ resp. $p^*$ between simplicial (co)homologies of the nerves $p_*: {\mathcal {H}}_*(X,A,{\mathcal {V}})\rightarrow {\mathcal {H}}_*(X,A,{\mathcal {U}})$ and $p^*: {\mathcal {H}}^*(X,A,{\mathcal {U}})\rightarrow {\mathcal {H}}^*(X,A,{\mathcal {V}})$. The Čech (co)homology is defined by $H_*(X,A):=\varprojlim _{{\mathcal {U}}} {\mathcal {H}}_*(X,A,{\mathcal {U}})$ and $H^*(X,A):=\varinjlim _{{\mathcal {U}}} {\mathcal {H}}^*(X,A,{\mathcal {U}})$.

Let $\phi \in H^*(X,A)$ and $\beta \in H_*(X,A\cup B)$ for some $A,B\subseteq X$. The direct limit can be defined as a disjoint union of all ${\mathcal {H}}^*(X,A,{\mathcal {U}})$ factored by the relation $\lambda -p^*(\lambda )$ and the inverse limit can be defined as $\{\mu \in \prod {\mathcal {H}}_*(X,A\cup B, {\mathcal {U}}):\,\,p_*(\mu _{{\mathcal {V}}})=\mu _{\mathcal {U}}\}$. Therefore, $\beta$ can be represented as a net $(\beta _{{\mathcal {U}}})_{\mathcal {U}}$ so that $\beta _{\mathcal {U}}=p_*\beta _{\mathcal {V}}$ for any refinement ${\mathcal {V}}$ of any covering ${\mathcal {U}}$, and $\phi$ can be represented by an element $\phi _{\tilde{{\mathcal {U}}}}$ contained in ${\mathcal {H}}^*(X,A,\tilde{{\mathcal {U}}})$ for some fine enough $\tilde{{\mathcal {U}}}$.

For each such $\tilde{{\mathcal {U}}}$, we define $\alpha _{\tilde{{\mathcal {U}}}}:=\phi _{\tilde{{\mathcal {U}}}} \frown \beta _{\tilde{{\mathcal {U}}}}\in {\mathcal {H}}_*(X,B,\tilde{{\mathcal {U}}})$ by means of simplicial cap product. If $\tilde{{\mathcal {V}}}$ is a refinement of $\tilde{{\mathcal {U}}}$, then the naturality of simplicial cap product—namely, the relation $\phi _{\tilde{{\mathcal {U}}}}\frown p_*(\beta _{\tilde{{\mathcal {V}}}})=p_*(p^*(\phi _{\tilde{{\mathcal {U}}}})\frown \beta _{\tilde{{\mathcal {V}}}})$—implies that $\alpha _{\tilde{{\mathcal {U}}}}=p_* \alpha _{\tilde{{\mathcal {V}}}}$. Therefore, we may consistently define $\alpha _{\mathcal {U}}$ for any ${\mathcal {U}}$ to be equal to $p_* \alpha _{{\mathcal {V}}}$ where ${\mathcal {V}}$ is a fine enough refinement of ${\mathcal {U}}$ such that $\alpha _{\mathcal {V}}$ is already defined. This construction yields an element $\alpha \in H_*(X,B)$ and we define the Čech cap product $\phi \frown \beta$ to be equal to $\alpha$.

Let $f: X\rightarrow X'$, $A,B\subseteq X$, $A', B'\subseteq X'$, $f(A)\subseteq A'$ and $f(B)\subseteq B'$. We will show the naturality of $\frown$, that is, the relation $\phi '\frown f_*(\beta )=f_*(f^*(\phi ')\frown \beta )$ for $\phi '\in H^*(X',A')$ and $\beta \in H_*(X, A\cup B)$.

Let ${\mathcal {U}}$ be a covering of *X* and ${\mathcal {U}}'$ a covering of $X'$ so that for any $U\in {\mathcal {U}}$ there exists $U'\in {\mathcal {U}}'$ such that $f(U)\subseteq U'$. Given $\mathcal {U'}$ and *f*, then any covering of *X* admits a refinement ${\mathcal {U}}$ with this property. Such map *f* and a choice of the set $U'$ for any *U* induces a simplicial map $f_\sharp : N({\mathcal {U}})\rightarrow N(\mathcal {U'})$; other assignment of the supersets $U'$ of *f*(*U*) yields a homotopic map. If ${\mathcal {V}}$ resp. ${\mathcal {V}}'$ is a refinement of ${\mathcal {U}}$ resp. ${\mathcal {U}}'$ such that *f* maps each $V\in {\mathcal {V}}$ into some $V'\in {\mathcal {V}}'$, then we have a square of simplicial maps between simplicial complexes

9

that commute up to homotopy and induce commuting maps on the level of their simplicial (co)homology. Thus, if $\beta \in H_*(X,B)$ is represented by $(\beta _{\mathcal {U}})_{\mathcal {U}}$, then $f_*v$ can be defined to by the net that assigns to each ${\mathcal {U}}'$ the $f_*$-image of $\beta _{{\mathcal {U}}}\in {\mathcal {H}}(X, A\cup B, {\mathcal {U}})$ where ${\mathcal {U}}$ is fine enough so that *f* maps elements of ${\mathcal {U}}$ to ${\mathcal {U}}'$. This is well defined, because if ${\mathcal {V}}$ is a refinement of ${\mathcal {U}}$, then the commutativity of the induced maps in the upper left triangle of (9) implies $f_* \beta _{\mathcal {V}}=f_* p_* \beta _{\mathcal {V}}=f_* \beta _{\mathcal {U}}$; further, any $\tilde{{\mathcal {U}}}$ such that *f* maps its element to elements of $\mathcal {U'}$ has a common refinement with ${\mathcal {U}}$. The commutativity of the induced maps in the lower right triangle of (9) implies that $p_* (f_* \beta )_{{\mathcal {V}}'}=(f_* \beta )_{{\mathcal {U}}'}$, hence $f_*\beta$ is a well defined element of $H_*(X,B)$.

Let $\phi '\in H^*(X',A')$ be represented by some $\phi _{\tilde{\mathcal {U'}}}'\in {\mathcal {H}}^*(X',A',\tilde{{\mathcal {U}}'})$. By construction, $\phi '\frown f_* \beta$ is represented by the net that assigns to any refinement $\tilde{{\mathcal {V}}'}$ of $\tilde{{\mathcal {U}}'}$ the cap product of

10 $$\begin{aligned} \phi _{\tilde{{\mathcal {V}}'}}'\frown f_*(\beta _{{\mathcal {U}}})\in {\mathcal {H}}_*(X',B', \tilde{{\mathcal {V}}'}), \end{aligned}$$

where ${\mathcal {U}}$ is fine enough so that *f* maps each $U\in {\mathcal {U}}$ to some ${\tilde{V}}'\in \tilde{{\mathcal {V}}'}$. It remains to show that $f_*(f^* \phi '\frown \beta )$ is represented by the same object. Let $\tilde{{\mathcal {V}}'}$ be a refinement of $\tilde{{\mathcal {U}}'}$, ${\mathcal {U}}$ be as in (10) and let ${\mathcal {V}}$ be a refinement of ${\mathcal {U}}$ fine enough so that *f* maps each of its sets to some element of $\tilde{{\mathcal {V}}'}$. The term $f_*(\beta _{\mathcal {U}})$ in (10) equals $f_* (\beta _{\mathcal {V}})$ and $f^*(\phi ')$ can be represented by $f^* (\phi _{\tilde{{\mathcal {V}}'}}')\in {\mathcal {H}}^*(X,A,{\mathcal {V}})$. It follows that $f_* (f^* \phi ' \frown \beta )$ can be represented by a net that assigns to $\tilde{{\mathcal {V}}'}$ the element $f_* (f^* (\phi _{\tilde{{\mathcal {V}}'}}')\frown \beta _{{\mathcal {V}}})\in {\mathcal {H}}_*(X',B',\tilde{{\mathcal {V}}'})$ which equals (10) by the naturality of simplicial cap product.

## Footnotes

1 Our spaces *K*, $Y'$ are denoted by “*X*, *A*” in the original references, but we changed this notation as we will use the symbols *X* and *A* for different purposes.

2 Authors of [4] develop a different notion of relativity that is based on considering a pair of spaces $(Y',Y'_0)$ instead of the single space $Y'$. This direction is rather orthogonal to the matters of this paper.

3 In [4, 11], well groups were defined by means of singular homology. But then, once we allow arbitrary continuous perturbations, to the best of our knowledge, no $f:K\rightarrow {\mathbb {R}}^n$ with nontrivial $U_j(f,r)$ for $j>0$ would be known. In particular, the main result of [4] would not hold. The correction via means of Steenrod homology was independently identified by the authors of [4].

4 Namely, so called *bottleneck distance* between a well diagrams of *f* and $f'$ is bounded by $\Vert f-f'\Vert$. The stability does not say how well the well diagrams describe the zero set. This question is also addressed in this paper.

5 The Poincaré dual of a cohomology class $\varphi$ can be in fact defined as the cap product $[X]\frown \varphi$, where [*X*] is so called fundamental homology class of the manifold *X*. One advantage of the cap product is that it is defined for any simplicial complex *X*.

6 We emphasize that the considered *r*-perturbations of *f* need not be neither simplexwise linear nor have rational values on the vertices.

7 We cannot even approximate the “robustness of roots”: it is undecidable, given a simplicial complex *K* and a simplexwise linear map $f:K\rightarrow {\mathbb {R}}^n$, whether there exists $\varepsilon >0$ such that $U_0(f,\varepsilon )$ is nontrivial or whether $U_0(f,1)$ is trivial. The extendability can always be decided for *n* even, however, the problem is less likely tractable for $\dim K>2n-2$.

8 The *i*-skeleton $X^{(i)}$ of a simplicial (cell) complex *X* is the subspace of *X* containing all simplices (cells) of dimension at most *i*.

9 This is the global description of the primary obstruction as presented in [32]. It can be shown that $\phi _f$ depends on the homotopy class of $f|_A$ only. Another way of defining the primary obstruction is the following. It is represented by the so-called *obstruction cocycle* $z_f\in Z^n(X,A)$ that assigns to each *n*-simplex $\sigma \in X$ the element $[f|_{\partial \sigma }]\in \pi _{n-1}(S^{n-1}) \cong {\mathbb {Z}}$ [28, Chap.  3]. Through this definition it is not difficult to derive that the map $f|_A$ can be extended to $X^{(n)}\rightarrow S^{n-1}$ if and only if $\phi _f=0$, see also [28, Chap. 3].

10 We give a precise definition of cap product between Čech homology and cohomology in Appendix 4.

11 This is the case for $f:{\mathbb {R}}^4\rightarrow {\mathbb {R}}^3$ given by $f(x):=|x|\eta (x/|x|)$ where $\eta :S^3\rightarrow S^2$ is the Hopf map.

12 Namely $U_2(f,r)\cong 0$ as is shown by the *r*-perturbation $g(x,y)=f(x,y)-rx$ with the zero set homeomorphic to the 3-sphere.

13 This dimension is somewhat important as all higher well groups are trivial by Lemma 7 and all lower homology groups of *X* may be trivial as is the case in Theorem 4. On the other hand, $H_{m-i}\big (X,\pi _{i-1}(S^{n-1})\big )$ has to be nontrivial in the case when *X* is a manifold for the reasons following from obstruction theory and Poincaré duality.

14 That is, there exist finite simplicial complexes $A^{\Delta }\subseteq X^{\Delta }$ and a homeomorphism $(X^{\Delta }, A^{\Delta } )\rightarrow (X,A).$

15 Here $[A_r,S^{n-1}]$ denotes the set of all homotopy classes of maps from $A_r$ to $S^{n-1}$, that is, the cohomotopy group $\pi ^{n-1}(A_r)$ when $\dim A_r\le 2n-4$.

16 Note that for $n=1$ the structure of the set $[A,S^{n-1}]$ is very simple and for $n=2$ we have $[A,S^{n-1}]\cong H^1(A;{\mathbb {Z}})$ no matter what the dimension of $A_r$ is.

17 The interleaving distance between the sequences $\pi ^{n-1}(A_r)$ and $\pi ^{n-1}(A'_r), r>0$ is at most $\alpha$ whenever there are families of homomorphisms of pointed Abelian groups $u_r:\pi ^{n-1}(A_r)\rightarrow \pi ^{n-1}(A'_{r+\alpha })$ and $v_r:\pi ^{n-1}(A'_r)\rightarrow \pi ^{n-1}(A_{r+\alpha })$ such that the compositions $v_{r+\alpha } u_r$ and $u_{r+\alpha } v_r$ give the corresponding inclusion induced maps.

18 We originally proved that when *K* is a triangulated orientable manifold, the Poincaré dual of $\phi _f$ is contained in $U_{m-n}(f,r)$. Expanding the proof was not difficult, but the preceding inspiration of replacing the Poincaré duality by cap product came from Patel. The cap product provides a nice generalization to an arbitrary simplicial complex *K*.

19 Naturality of the cap product means that if $f: (X,A\cup B,A)\rightarrow (X;A'\cup B',A')$ is continuous, then $f_*(f^*({{\tilde{\alpha }}})\frown \beta )={{\tilde{\alpha }}}\frown f_*(\beta )$ for any $\beta \in H_*(X,A\cup B)$ and ${{\tilde{\alpha }}}\in H^*(X',A')$.

20 Because of our careful choice of the spaces $A_i$ and $A'_i$ we do not need the strong excision here. However, we do not know how to avoid it in the case when $\Vert g-f\Vert =r$.

21 We assume that, for simplicial (co)homology computations, an orientation of all simplices had been chosen. The notation $f'(\tau ^n)=\pm \sigma ^n$ means that $f'$ maps vertices of $\tau ^n$ onto vertices of $\sigma ^n$ in an orientation preserving/reversing way.

22 Thus the formal definition is $e'({\varPsi }_1(z)):=|z|e'\big ({\varPsi }_1(z/|z|)\big )$.

23 With more effort we could show that for *any* point *z* of *X* there is an *r*-perturbation of $f'$ with *z* being its only zero point.

24 The only remaining category consists of those $f''$ where $f''|_A$ extends to whole of *X*, i.e., $U_*(f,r)\cong 0$, or equivalently, $\emptyset \in Z_r(f)$.
